# An Improved Similarity-Based Clustering Algorithm for Multi-Database Mining

**DOI:** 10.3390/e23050553

**Published:** 2021-04-29

**Authors:** Salim Miloudi, Yulin Wang, Wenjia Ding

**Affiliations:** School of Computer Science, Wuhan University, Wuhan 430072, China

**Keywords:** coordinate descent, clustering, multi-database mining, fuzziness, binary entropy loss, similarity matrix

## Abstract

Clustering algorithms for multi-database mining (MDM) rely on computing $(n^{2}-n)/2$ pairwise similarities between *n* multiple databases to generate and evaluate $m\in [1,\left(n^{2}-n\right)/2]$ candidate clusterings in order to select the ideal partitioning that optimizes a predefined goodness measure. However, when these pairwise similarities are distributed around the mean value, the clustering algorithm becomes indecisive when choosing what database pairs are considered eligible to be grouped together. Consequently, a trivial result is produced by putting all the *n* databases in one cluster or by returning *n* singleton clusters. To tackle the latter problem, we propose a learning algorithm to reduce the fuzziness of the similarity matrix by minimizing a weighted binary entropy loss function via gradient descent and back-propagation. As a result, the learned model will improve the certainty of the clustering algorithm by correctly identifying the optimal database clusters. Additionally, in contrast to gradient-based clustering algorithms, which are sensitive to the choice of the learning rate and require more iterations to converge, we propose a learning-rate-free algorithm to assess the candidate clusterings generated on the fly in fewer upper-bounded iterations. To achieve our goal, we use coordinate descent (CD) and back-propagation to search for the optimal clustering of the *n* multiple database in a way that minimizes a convex clustering quality measure L(θ) in less than $(n^{2}-n)/2$ iterations. By using a max-heap data structure within our CD algorithm, we optimally choose the largest weight variable $\theta_{p,q}^{\left(i\right)}$ at each iteration *i* such that taking the partial derivative of L(θ) with respect to $\theta_{p,q}^{\left(i\right)}$ allows us to attain the next steepest descent minimizing L(θ) without using a learning rate. Through a series of experiments on multiple database samples, we show that our algorithm outperforms the existing clustering algorithms for MDM.

## 1. Introduction

Large multi-branch companies need to analyze multiple databases to discover useful patterns for the decision-making process. To make global decisions for the entire company, the traditional approach suggests to merge and integrate the local branch-databases into a huge data warehouse, and then one can apply data mining algorithms [1] to the accumulated dataset to mine the global patterns useful for all the branches of the company. However, there are some limitations associated with this approach. For instance, the cost of moving the data over the network, and integrating and storing potentially heterogeneous databases could be expensive. Moreover, some branches may not accept sharing their raw data due to the underlying privacy issues. More crucially, integrating a large amount of irrelevant data can easily disguise some essential patterns hidden in multiple databases. To tackle the latter problems, it is suggested to keep the transactional data stored locally and only forward the local patterns mined at each branch database to a central site where they will be clustered into disjoint cohesive pattern-base groups for knowledge discovery. In fact, analyzing the local patterns present in each individual cluster of the multiple databases (MDB) enhances the quality of aggregating novel relevant patterns, and also facilitates the parallel maintenance of the obtained database clusters.Various clustering algorithms and models have been introduced in the literature, namely spectral-based models [2], hierarchical [3], partitioning [4], competitive learning-based models [5,6,7] and artificial neural networks (ANNs) based clustering [8,9,10]. Additionally, clustering could be applied in many domains [11,12] including community discovery in social networks [13,14], image segmentation [15,16] and recommendation systems [17,18,19]. In this article, we focus on exploring similarity-based clustering models for multi-database mining [20,21,22,23], due to their stability, simplicity [24] and robustness in partitioning graphs of *n* multiple databases into *k* connected components consisting of similar database objects. Nevertheless, the existing clustering quality measures in [20,21,22,23] are non-convex objectives suffering from the existence of local optima. Consequently, identifying the optimal clustering may be a difficult task, as it requires evaluating all the candidate clusterings generated at all the local optima in order to find the ideal clustering.

To address the issues associated with clustroid initialization, preselection of a suitable number of clusters and non-convexity of the clustering quality objectives, we proposed in [25,26] an algorithm named GDMDBClustering, which minimizes a quasi-convex loss function quantifying the quality of the multi-database clustering, without a priori assumptions about which number of clusters should be chosen. Therefore, in contrast to the clustering models proposed in [20,21,22,23], GDMDBClustering [25] does not require us to produce and assess all the possible candidate classifications in order to find the optimal partitioning. Alternatively, each partitioning is assessed on the fly as it is generated and the clustering algorithm terminates right after attaining the global minimum of the objective function. However, the existing gradient-based clustering algorithms [25,26] are strongly dependent on the choice of the learning rate η, which influences the number of learning cycles required to find the optimal partitioning. In fact, selecting a larger η value may cause global minimum overshooting and setting a smaller η value may necessitate many learning iterations for the algorithm to converge.

In this paper, we improve upon previous work [25,26] and propose a learning-rate-free (i.e., independent of the learning rate η) algorithm requiring fewer upper-bounded iterations (i.e., the maximum number of iterations is at most $(n^{2}-n)/2$) to minimize a clustering convex loss function L(θ) using coordinate descent (CD) and back-propagation. Precisely, our proposed algorithm minimizes a quadratic hinge-based loss L(θ) over the first largest coordinate variable $\theta_{p,q}$ while keeping the rest of the $(\frac{n}{2})-1$ variables fixed. Then, it minimizes L(θ) over the second largest coordinate variable while keeping the rest of the $(\frac{n}{2})-1$ variables fixed, and so on until convergence or until cycling through all the $\left(\frac{n}{2}\right)$ coordinate variables. Consequently, our algorithm becomes faster than GDMDBClustering [25] which is dependent on a learning rate and also requires us to minimize the cost over a large set of variables at each iteration. This can be a very challenging problem in contrast to minimizing the loss over one single variable at a time while keeping all the other dimensions fixed.

On the other hand, existing clustering algorithms for multi-database mining (MDM) [20,21,22,23,25,26] proceed by computing $(n^{2}-n)/2$ pairwise similarities $sim\left(D_{p},D_{q}\right)\in \left[0,1\right]$ between *n* multiple databases, and then use these values to generate and evaluate $m\in [1,\left(n^{2}-n\right)/2]$ candidate clusterings in order to select the ideal partitioning optimizing a given goodness measure. However, when $sim{\left(D_{p},D_{q}\right)}_{n\times n}$ (p=0,…,n−2,q=p+1,…,n−1) are distributed around the mean value μ=0.5, the fuzziness index of the similarity matrix increases and the clustering algorithm becomes uncertain when choosing what database pairs are considered similar and hence eligible to be put into the same cluster. Consequently, a trivial result is produced, i.e., putting all the *n* databases in one cluster or returning *n* singleton clusters. To tackle the latter problem, we propose a learning algorithm to reduce the fuzziness in the pairwise similarities by minimizing a weighted binary entropy loss function H(·) via gradient descent and back-propagation. Precisely, the learned model will force the similarity values above 0.5 to go closer to their maximum value (≈1), and let those below 0.5 go closer to their minimum value (≈0) in a way that minimizes H(·). This will significantly reduce the associated fuzziness and improve the certainty of the clustering algorithm to correctly identify the optimal database clusters. The main contributions of this article are listed as follows:

- Unlike the existing algorithms proposed in [20,21,22,23,25,26] where one-class trivial clusterings are produced when the similarity values are centered around the mean value, we have added a preprocessing layer prior to clustering where the pairwise similarities are adjusted to reduce the associated fuzziness and hence improve the quality of the produced clustering. Our experimental results show that reducing the fuzziness of the similarity matrix helps generating meaningful relevant clusters that are different to the one-class trivial clusterings.
- Unlike the multi-database clustering algorithms proposed in [20,21,22,23], our approach uses a convex objective function L(θ) to assess the quality of the produced clustering. This allows our algorithm to terminate just after attaining the global minimum of the objective function (i.e., after exploring fewer similarity levels). Consequently, this avoids generating unnecessary candidate clusterings, and hence reduces the CPU overhead. On the other hand, the clustering algorithms in [20,21,22,23] use non-convex objectives (i.e., they suffer from the existence of local optima due to the use of more than two monotonic functions), and therefore require generating and evaluating all the $(n^{2}-n)/2$ local candidate clustering solutions in order to find the clustering located at the global optimum.
- Furthermore, unlike the previous gradient-based clustering algorithms [25,26], our proposed algorithm is leaning-rate-free (i.e., independent of the learning rate), and needs at most (in the worst case) $(n^{2}-n)/2$ iterations to converge. That is why our proposed algorithm is faster than GDMDBClustering [25], which is strongly dependent on the learning step size η and its decay rate.
- Additionally, unlike the similarity measure proposed in [20], which assumes that the same threshold was used to mine the local patterns from the *n* transactional databases, our proposed similarity measure takes into account the existence of *n* different local thresholds, which are then combined to calculate a new threshold for each cluster. Afterward, using the new thresholds, our similarity measure accurately estimates the valid patterns post-mined from each cluster in order to compute the $(n^{2}-n)/2$ pairwise similarities.
- The experiments carried out on real, synthetic and randomly generated datasets show that the proposed clustering algorithm outperforms the compared clustering models in [20,21,22,23,25,26], as it has the shorted average running time and the lowest average clustering error.

The remainder of this paper is organized as follows: Section 2 presents an example motivating the importance of clustering for multi-database mining (MDM) and also reviews traditional clustering algorithms for MDM. Section 3 defines the main concepts related to similarity-based clustering and then introduces the proposed approach and its main components. Section 4 presents and analyzes the experimental results. Finally, Section 5 draws conclusions and highlights potential future work.

## 2. Motivation and Related Work

### 2.1. Motivating Example

Prior to mining the multiple databases (MDB) of a multi-branch enterprise, it is essential to cluster these MDB into disjoint and cohesive pattern-base groups sharing an important number of local patterns in common. Then, using local pattern analysis and pattern synthesizing techniques [27,28,29,30], one can examine the local patterns in each individual cluster to discover novel patterns, including the *exceptional patterns* [31] and the *high-vote patterns* [32], which are extremely useful when it comes to making special targeted decisions regarding each cluster of branches of the same corporation. In the following example, we show the impact of clustering the multi-databases of a multi-branch corporation prior to multi-database mining. Consider the six transactional databases $D=\cup_{p=1}^{6}\left\{D_{p}\right\}$ shown in Table 1, where each database $D_{p}$ records a set of transactions enclosed in parentheses and each transaction contains a set of items separated by commas. Consider a minimum support threshold α=0.5. The local frequent itemsets, denoted by $FIS(D_{p},\alpha )$, and discovered from each database $D_{p}$ are shown in Table 2, such that $I_{k}$ in each tuple $\langle I_{k},supp\left(I_{k},D_{p}\right)\rangle$ of $FIS(D_{p},\alpha )$ is the frequent itemset name and $supp(I_{k},D_{p})$, named support, is the ratio of the number of transactions in $D_{p}$ containing $I_{k}$ to the total number of transactions in $D_{p}$.

Now, the global support of each itemset $I_{k}\in \cup_{p=1}^{6}\left\{FIS\left(D_{p},0.5\right)\right\}$ is calculated via the synthesizing equation [33] defined as follows:

(1) $$\begin{array}{cc} supp(I_{k},D) & =\frac{\sum_{p=1}^{n}\left|D_{p}\right|\times supp\left(I_{k},D_{p}\right)}{\sum_{p=1}^{n}\left|D_{p}\right|} \end{array}$$

where n=6 is the total number of databases in D and $|D_{p}|$ is the number of transactions in $D_{p}$. For instance, we can calculate the global support of the itemset *A* as follows:

$$\begin{array}{cc} supp(A,D) & =\frac{0.75\times 4+0.8\times 5+0.5\times 4+0\times 3+0\times 4+0\times 4}{4+5+4+3+4+4}\\ & =0.375<\alpha \end{array}$$

After computing the global supports of the rest of the itemsets using (1), no single novel pattern has been found, i.e., $\forall \;I_{k}\in \cup_{p=1}^{6}\left\{FIS\left(D_{p},0.5\right)\right\},supp\left(I_{k},D\right)<0.5$). The reason is that irrelevant patterns were involved in the synthesizing procedure. Now, if we examine the frequent itemsets in Table 2, we observe that some databases share many patterns in common. Precisely, the six databases seem to form two clusters, $C_{1}$ = $\left\{D_{1},D_{2},D_{3}\right\}$ and $C_{2}$ = $\left\{D_{4},D_{5},D_{6}\right\}$, where each cluster of databases tend to share similar frequent itemsets.

Next, let us use the synthesizing Equation (1) on the frequent itemsets coming from every single cluster $C_{i}$, such that 4≤p≤6=n for cluster $C_{2}$ and 1≤p≤3=n for cluster $C_{1}$. This time, new valid frequent itemsets having a support value above the minimum threshold α are discovered in the two clusters. In fact, $FIS(C_{2},0.5)$ = {〈FH,0.727〉,〈F,0.727〉,〈H,0.818〉} and $FIS(C_{1},0.5)$ = {〈C,0.769〉,〈B,0.769〉,〈A,0.692〉}. The obtained patterns show that a percentage of more than 69% of the total transactions in the cluster $C_{1}$ include the itemsets *C*, *B* and *A*. More than 72% of the total transactions in the cluster $C_{2}$ include FH, *F* and *H*. Moreover, some associations between itemsets could be derived as well, such that the itemset $\langle F\;H,0.727\rangle \in FIS\left(C_{2},0.5\right)$ suggests that on average, if a customer collects the item *H* at one of the branches in $C_{2}$, they are likely to also buy the item *F* with a $\frac{supp(FH,C_{2})}{supp(H,C_{2})}=88.87\%$ confidence.

The above example demonstrates the importance of clustering the multi-databases into disjoint cohesive clusters before synthesizing the global patterns. In fact, when the local patterns mined from the six databases were analyzed all together, no global pattern could be synthesized. On the other hand, when the six databases were divided into two different clusters and then each cluster was analyzed individually, useful and novel patterns (knowledge) were discovered. Actually, from the discovered knowledge, decision makers and stakeholders are going to have a clear vision about the branches that exhibit similar purchasing behaviors, and hence take useful decision accordingly. In fact, appropriate business decisions may be taken regarding each group of similar branches in order to predict potential purchasing patterns, increase the customer retention rate and convince customers to purchase more services in the future. Consequently, exploring and examining individual clusters of similar local patterns is going to help the discovery of new and relevant patterns capable of improving the decision-making quality.

### 2.2. Prior Work

The authors in [34] have adopted a divide and conquer *mono-database mining* approach to accelerate mining global frequent itemsets (FIs) in large transactional databases. In [35,36], the authors have proposed similar work where big transactional databases are divided into *k* disjoint transaction partitions whose sizes are small enough to be read and loaded to the random access memory. Then, the frequent itemsets (FIs) mined from all the *k* partitions are synthesized into global FIs using an aggregation function such as the one suggested by the authors in [33]. It is worth noting that for mono-database mining applications, we usually have direct access to the raw data stored in big transactional databases. On the other hand, for multi-database mining (MDM) applications, it is suggested to keep the transactional data stored locally and only forward the local patterns mined at each branch database to a central site where they will be clustered into disjoint cohesive pattern-base groups for knowledge discovery. As a result, the confidential raw data are kept safe, and also the cost associated with transmitting a large amount of data over the network is cut off. Hence, in contrast to clustering the transactional data stored in a single data warehouse, our approach consists of clustering the local patterns mined and forwarded from multi-databases without requiring the number of clusters to be set a priori. Our purpose is to identify the group of databases that share similar patterns, such as the *high-vote patterns* [32] and the *exceptional patterns* [31,37,38] that can be used to make specific decisions regarding their corresponding branches. In the traditional clustering approach [34,35,36] applied for mono-database mining, we can only mine the global patterns that are supported by the whole multi-branch company.

The existing clustering algorithms for multi-database [20,21,23,39,40] are based on an agglomerative process that generates hierarchical partitionings at different levels of similarity, where each cluster in a given candidate partitioning is included in another cluster of a partitioning produced at the next similarity level. Regardless of the latter observation, each candidate partitioning is produced without taking into account the use of the clusters generated at the previous similarity levels. As a result, the clustering algorithms in [20,21,23,39,40] unnecessarily reconstruct clusters that have been built at the previous similarity levels. The latter limitation inspired the authors in [22] to design a graph-based algorithm, which maintains the classes produced at prior similarity levels in order to produce new subsequent classes out of them. Despite the fact that the experiments done in [22] showed promising results against the prior work [20,21,23,39,40], these algorithms are based on non-convex functions to evaluate the quality of the produced candidate clusterings. Consequently, finding the ideal clustering for which a non-convex function is optimal may be a difficult problem to solve in a short time.

To face the latter problem, the authors in [26] have transformed the clustering problem into a quasi-convex optimization problem solvable via gradient descent and back-propagation. Consequently, an early stopping of the clustering process occurs right after converging to the global minimum. Hence, by avoiding generating and evaluating unnecessary candidate clusterings, we can significantly reduce the CPU execution time. Even though traditional clustering algorithm such as k-means [4,41] are intuitive, popular and not hard to implement, they remain sensitive to clustroid initialization, preselection of a suitable number of clusters and non-convexity of the clustering quality objective [42]. The silhouette plot [43] could be used to find an appropriate number of clusters, but this requires executing k-means multiple times with different number of clusters in order to find the ideal partitioning maximizing the silhouette objective. As a result, the time performance will be influenced in the case of clustering big high-dimensional datasets. Slightly different, hierarchical-based clustering algorithms [3] build nested hierarchical levels to visualize the relationships between different objects in the form of dendrograms. Then, it is up to the domain expert or to some non-convex metrics to determine at which level the tree diagram should be cut.

Conversely, the optimization problem formulated in [25,26] is quasi-convex. Therefore, convergence to the global optimum is independent of the initial settings. Furthermore, the proposed gradient-based clustering GDMDBClustering [25] does not need to have the number of clusters as a parameter. Alternatively, the number of clusters becomes a parametric function in the main objective. However, GDMDBClustering is based on the choice of a suitable learning rate, i.e., choosing a small learning rate η may increase the number of iterations and slow down learning the optimal weights, whereas a large η may let the algorithm overshoot the global minimum. To overcome the latter limitation, we propose in this paper a learning-rate-free clustering algorithm, named CDClustering, which minimizes a convex objective function quantifying the clustering quality. For this purpose, we use coordinate descent (CD) and back-propagation to search for the optimal clustering of *n* multiple database in less than $(n^{2}-n)/2$ iterations and without using a learning rate. This makes our algorithm faster than the previous gradient-based clustering algorithm [25,26] which remains dependent on a learning rate defined based on some prior knowledge of the properties of the loss function. On the other hand, due to the fuzziness of the similarity matrix, which increases when the pairwise similarities are distributed around the mean value, the clustering algorithm becomes indecisive when grouping similar databases together. To face this problem, we design a learning algorithm to adjust the pairwise similarities between *n* multiple databases, in a way which minimizes a binary entropy loss function quantifying the fuzziness associated with the similarity matrix. Thus, the proposed algorithm becomes crisp in discriminating between the different database clusters.

## 3. Materials and Methods

In this section, we present our fuzziness reduction model applied to the pairwise similarities between *n* multiple databases and describe in details our coordinate descent-based clustering approach. Some definitions and notions relevant to this work need to be presented first.

### 3.1. Background and Relevant Concepts

In this subsection, we define the similarity measure between two transaction databases and present the process of generating and evaluating a given candidate clustering. We also define four clustering validity functions used to evaluate the clustering quality.

#### 3.1.1. Similarity Measure

Each transactional database $D_{p}$ is encoded as a hash-table to be defined as follows:

(2) $$FIS\left(D_{p},\alpha_{p}\right)=\left\{\cup_{k=1}^{m}\langle I_{k},supp\left(I_{k},D_{p}\right)\rangle \;|\;supp\left(I_{k},D_{p}\right)\geq \alpha_{p}\right\}$$

where p=0,…,n−1, *n* is the number of transactional databases, *m* is the number of frequent itemsets in $D_{p}$, $I_{k}$ is the name of the *k*-th frequent itemset, $supp\left(I_{k},D_{p}\right)\in \left[0,1\right]$ is the support of $I_{k}$, which is the ratio of the number of rows in $D_{p}$ containing $I_{k}$ to the total number of rows in $D_{p}$, and $\alpha_{p}\in \left[0,1\right]$ is the minimum support threshold corresponding to $D_{p}$, such that $supp\left(I_{k},D_{p}\right)\geq \alpha_{p}$. In this paper, FP-Growth [1] algorithm is used to mine the frequent itemsets in each database $D_{p}$ as it only requires two passes over the whole database. Our proposed similarity measure is based on maximizing the number of global frequent itemsets (FIs) synthesized from the local FIs in each cluster. Precisely, to measure the similarity between two transactional databases $D_{q}$ and $D_{p}$, for p=0,…,n−2, q=p+1,…,n−1, we define the following function:

(3) sim(Dp,Dq)=∑Ψ(Ik,{Dp,Dq})k,Ik∈{FIS(Dp,αp)∩FIS(Dq,αq)}|FIS(Dp,αp)∪FIS(Dq,αq)|

where

(4) $$\begin{array}{c} \Psi \left(I_{k},\left\{D_{p},D_{q}\right\}\right)=\left\{\begin{array}{c} 1\;,\;\mathrm{if}\;supp\left(I_{k},\left\{D_{p},D_{q}\right\}\right)\geq \alpha_{p,q}\\ 0\;,\mathrm{otherwise} \end{array}\right. \end{array}$$

such that

(5) $$\begin{array}{c} supp\left(I_{k},\left\{D_{p},D_{q}\right\}\right)=\frac{supp\left(I_{k},D_{p}\right)\times |D_{p}|+supp\left(I_{k},D_{q}\right)\times \left|D_{q}\right|}{|D_{p}\left|+\right|D_{q}|} \end{array}$$

and

(6) $$\begin{array}{c} \alpha_{p,q}=\frac{\alpha_{p}\times |D_{p}|+\alpha_{q}\times \left|D_{q}\right|}{|D_{p}\left|+\right|D_{q}|} \end{array}$$

We note that the operator |·| is the cardinality of the set passed in as argument. Multiplying $\alpha_{p}$ by $|D_{p}|$ returns the minimum number of transactions in which a frequent itemset $I_{k}$ should occur in $D_{p}$. Therefore, $\alpha_{p,q}$ is the minimum percentage of transactions from the cluster $C_{p,q}=\left\{D_{p},D_{q}\right\}$ containing the itemset $I_{k}$, i.e., $supp\left(I_{k},C_{p,q}\right)\geq \alpha_{p,q}$. In fact, the similarity measure sim in Formula (3) takes into account the local minimum support threshold at each database to calculate a new threshold for each cluster. In this paper, instead of writing ’the similarity measure sim in Formula (3)’, we often write sim (3).

#### 3.1.2. Clustering Generation and Evaluation

Let $C(D,\delta_{i})$ = $\left\{C_{1},C_{2},\ldots ,C_{k}\right\}$ be a candidate clustering of D = $\left\{D_{0},D_{1},\ldots ,D_{n-1}\right\}$ produced at a given level of similarity $\delta_{i}\in \left[0,1\right]$, such that $\cap_{j=1}^{k}\left\{C_{j}\right\}=\emptyset$ and $\cup_{j=1}^{k}\left\{C_{j}\right\}=D$. From a graph-theoretic perspective, each cluster $C_{j}$ represents a connected component in a similarity graph G=(D,E), and an edge $\left(D_{p},D_{q}\right)$ is added to the list of edges *E* if and only if $sim\left(D_{p},D_{q}\right)\geq \delta_{i}$, where p=0,…,n−2,q=p+1,…,n−1.

Initially, G=(D,E) has no edge, i.e., E=∅. Then, at a given similarity level $\delta_{i}\in \left[0,1\right]$, edges $\left(D_{p},D_{q}\right)$ satisfying $sim\left(D_{p},D_{q}\right)\geq \delta_{i}$, are added to *E*. The level of similarity $\delta_{i}$ (i=1,…,m) is chosen from the list of the *m* unique sorted pairwise similarities $sim(D_{p},D_{q})$ computed between the *n* transaction databases, such that $\delta_{1}>\delta_{2}>\ldots \delta_{i-1}>\delta_{i}>\delta_{i+1}>\ldots >\delta_{m}$ and $m\leq (n^{2}-n)/2$. After adding all the edges $\left(D_{p},D_{q}\right)$ at $\delta_{i}$, each graph component $C_{j}$(j=1,…,k) will be representing one database cluster in our candidate partitioning $C(D,\delta_{i})$. One can then use one of the clustering goodness measures shown in Table 3 to assess the quality of $C(D,\delta_{i})$.

Once we generate and evaluate all the $m\leq (n^{2}-n)/2$ candidate clusterings, we report the global optimum (minimum or maximum) of the goodness measure and compare its corresponding clustering with the ground truth if it is known or with the clustering generated at the maximum point of the silhouette coefficient when the ground truth is unknown. In fact, the silhouette coefficient SC(D)∈[−1,1] proposed in [43,44] (See the last row in Table 3) could be used to verify the correctness of the cluster labels assigned to the *n* transactional databases. Precisely, a value SC(D)≈1 suggests that the *n* transactional databases are highly matched to their own clusters and loosely matched to their neighboring clusters.

We should note that each clustering goodness measure in Table 3 depends on more than two monotonic functions. For instance, the quality measure goodness (see the first row in Table 3) proposed in [20] is based on maximizing both the intra-cluster similarity W(D) (which is a non-decreasing function on the interval [0,1]) and the inter-cluster distance B(D) (which is a non-increasing function on the interval [0,1]), while minimizing the number of clusters f(D) (which is a non-increasing function on the interval [0,1]). Consequently, as it was shown via the experiments done in [25,26], most of the time, the graphs of the objectives functions in Table 3 show a non-convex behavior, which makes identifying the ideal partitioning a hard problem to solve without generating and evaluating all the candidate clusterings generated at the local optima.

### 3.2. Similarity Matrix Fuzziness Reduction

In this subsection, we present our fuzziness reduction model applied to the pairwise similarities between *n* multiple databases. Let $z_{p,q}=\theta_{p,q}\times x_{p,q}$ be a weighted similarity, such that $x_{p,q}=sim\left(D_{p},D_{q}\right)$ is the similarity value between $D_{p}$ and $D_{q}$ using Formula (3) and $\theta_{p,q}$ is the weight value associated with $x_{p,q}$ where p=0,…,n−2,q=p+1,…,n−1. Let g:R→]0,1[ be a continuous piecewise linear activation function and ∂g be its partial derivative defined as follows:

(7) $$\begin{array}{c} g\left(z_{p,q},\epsilon \right)=\max\left(z_{p,q},\epsilon \right)-\frac{\mathrm{sgn}(z_{p,q}-1+\epsilon )+1}{2}\;\left(z_{p,q}-1+\epsilon \right) \end{array}$$

(8) $$\begin{array}{c} \frac{\partial g(z_{p,q},\epsilon )}{\partial z_{p,q}}=\frac{\mathrm{sgn}(z_{p,q}-\epsilon )+1}{2}-\frac{\mathrm{sgn}(z_{p,q}-1+\epsilon )+1}{2} \end{array}$$

**Figure 1:**
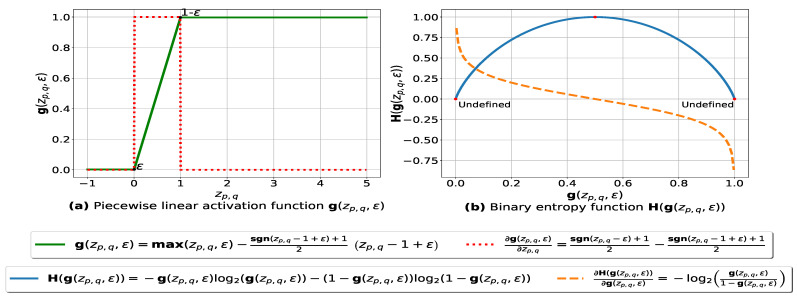
(**a**): represents (in green) the graph of the piecewise linear activation function g(·) and (in red) its partial derivative. We note that $z_{p,q}=\theta_{p,q}\times x_{p,q}$, and $\theta_{p,q}$ is the weight associated with the similarity value $x_{p,q}=sim\left(D_{p},D_{q}\right)$, sgn:R→{−1,1} is the signum function and ϵ is a small number (≈1e–7) ensuring that $g\left(z_{p,q},\epsilon \right)$ is always above 0 and below 1. (**b**): represents the binary entropy function H:(0,1)→(0,1] in blue and its partial derivative in orange.

The graph plots of $g(z_{p,q},\epsilon )$ and $\frac{\partial g(z_{p,q},\epsilon )}{\partial z_{p,q}}$ with respect to $z_{p,q}$ are depicted in Figure 1a. The parameter ϵ ensures that each value $z_{p,q}$ is within the range [ϵ,1−ϵ] such that ϵ is a very small number (e.g., ϵ=1e–7) forcing $g\left(z_{p,q},\epsilon \right)$ to be always above 0 and below 1, so that it can be plugged into our log-based loss function defined in (10).

#### 3.2.1. Fuzziness Index

The fuzziness index of the pairwise similarity vector $X^{T}$ = $\left[sim\left(D_{0},D_{1}\right),sim\left(D_{0},D_{2}\right),\ldots ,sim\left(D_{n-2},D_{n-1}\right)\right]$, also known as the entropy of the fuzzy set $X^{T}$ [45], and defined from Rn2 to [0,1], is given as follows:

(9) $$\begin{array}{cc} Fuzziness(X) & =\frac{-2}{n^{2}-n}\left(X^{T}\cdot \log_{2}\left(X\right)+\left(1-X^{T}\right)\cdot \log_{2}\left(1-X\right)\right)\\ & =\frac{-2}{n^{2}-n}\sum_{p=0}^{n-2}\sum_{q=p+1}^{n-1}\left(sim\left(D_{p},D_{q}\right)\log_{2}\left(sim\left(D_{p},D_{q}\right)\right)+\left(1-sim\left(D_{p},D_{q}\right)\right)\log_{2}\left(1-sim\left(D_{p},D_{q}\right)\right)\right) \end{array}$$

The smaller the value of Fuzziness(X), the better the clustering performance, and vice-versa. In fact, reducing the fuzziness of the pairwise similarities will lead to a more crisp decision making when it comes to finding the optimal partitioning of the *n* multiple databases. Particularly, the fuzziness of the similarity matrix increases when the pairwise values are centered around 0.5, resulting in more confusion when we need to decide whether two databases should be in the same cluster or not.

#### 3.2.2. Proposed Model and Algorithm

To reduce the fuzziness associated with the $(n^{2}-n)/2$ pairwise similarities between the *n* transaction databases D = $\left\{D_{0},D_{1},\ldots ,D_{n-1}\right\}$, we need to make the similarity values that are above the mean value μ=0.5 go closer to 1, and adjust the similarity values that are below μ=0.5 to go closer to 0. To do so, we consider the minimization of the sum of the binary entropy loss functions over the $(n^{2}-n)/2$ weighed similarity values $z_{p,q}=\theta_{p,q}\times x_{p,q}$ as follows:

(10) $$\begin{array}{cc} \arg\min_{\theta }\;H\left(\theta ,\epsilon \right) & \;=\;\arg\min_{\theta }\;\frac{2}{n^{2}-n}\sum_{p\;=\;0}^{n\;-\;2}\sum_{q\;=\;p\;+\;1}^{n\;-\;1}H\left(g\left(z_{p,q},\epsilon \right)\right)\\ & \;=\;\arg\min_{\theta }\;\frac{-2}{n^{2}-n}\sum_{p\;=\;0}^{n\;-\;2}\sum_{q\;=\;p\;+\;1}^{n\;-\;1}\left(g\left(z_{p,q},\epsilon \right)\log_{2}\left(g\left(z_{p,q},\epsilon \right)\right)+\left(1\;-\;g\left(z_{p,q},\epsilon \right)\right)\log_{2}\left(1\;-\;g\left(z_{p,q},\epsilon \right)\right)\right)\\ & \;=\;\arg\min_{\theta }\;\frac{-2}{n^{2}-n}\left(g\left(\theta^{T}⊙X^{T},\epsilon \right)\cdot \log_{2}\left(g\left(\theta ⊙X,\epsilon \right)\right)+\left(1\;-\;g\left(\theta^{T}⊙X^{T},\epsilon \right)\right)\cdot \log_{2}\left(1\;-\;g\left(\theta ⊙X,\epsilon \right)\right)\right) \end{array}$$

such that *n* is the number of databases, $\theta^{T}=\left[\theta_{0,1},\theta_{0,2},\ldots ,\theta_{n-2,n-1}\right]$ represents the model weight vector, $z_{p,q}$ represents the weighted similarity $\theta_{p,q}\times sim\left(D_{p},D_{q}\right)$ and $g(z_{p,q},\epsilon )$ is the activation function defined in (7). The graph plots of $H(g\left(z_{p,q},\epsilon \right))$ and $\frac{\partial H(g\left(z_{p,q},\epsilon \right))}{\partial g(z_{p,q},\epsilon )}$ with respect to $g(z_{p,q},\epsilon )$ are depicted in Figure 1b. Since the fuzziness of the similarity matrix is influenced by the weights associated with the pairwise similarities, the degree to which a pair of databases $\left(D_{p},D_{q}\right)$ belongs to the same cluster could be changed by adjusting the corresponding weight $\theta_{p,q}$, which is learned by minimizing (10) via gradient descent and back-propagation. The training equations are derived as follows:

(11) $$\theta_{p,q}=\theta_{p,q}-\eta \frac{\partial H(\theta ,\epsilon )}{\partial \theta_{p,q}}$$

where

(12) $$\begin{array}{cc} \frac{\partial H(\theta ,\epsilon )}{\partial \theta_{p,q}} & =\frac{-2}{n^{2}-n}\frac{\partial g(z_{p,q},\epsilon )}{\partial z_{p,q}}\frac{\partial z_{p,q}}{\partial \theta_{p,q}}\log_{2}\left(\frac{g(z_{p,q},\epsilon )}{1-g(z_{p,q},\epsilon )}\right)\\ \; & =\frac{-2}{n^{2}-n}\frac{\partial g(z_{p,q},\epsilon )}{\partial z_{p,q}}x_{p,q}\log_{2}\left(\frac{g(z_{p,q},\epsilon )}{1-g(z_{p,q},\epsilon )}\right) \end{array}$$

Let $\eta_{0}$ and epochs be the initial learning rate and the maximum number of learning iterations, respectively. At each epoch *i*, the current learning rate η decreases as follows:

(13) $$\begin{array}{c} \eta =\eta_{0}\times \left(1-i/epochs\right) \end{array}$$

**Figure 2:**
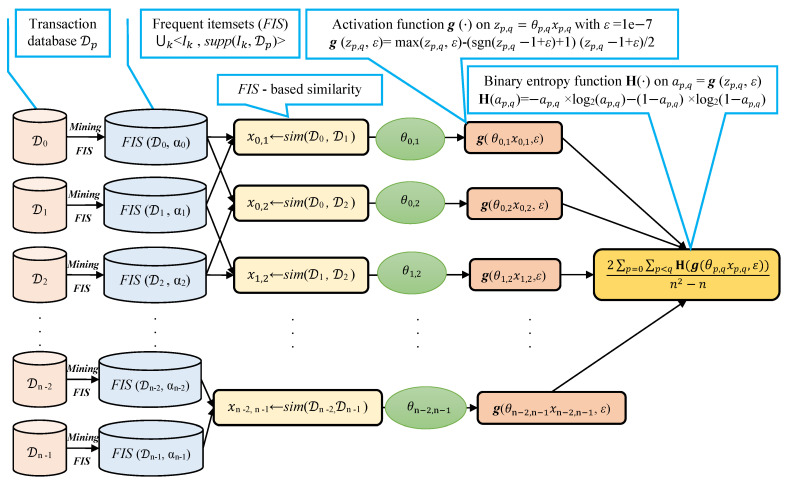
Proposed fuzziness reduction model on the $(n^{2}-n)/2$ pairwise similarities $x_{p,q}=sim\left(D_{p},D_{q}\right)$, p=0,…,n−2,q=p+1,…,n−1. We note that the graphs corresponding to the activation function g(·) and the binary entropy function H(·) are plotted in Figure 1.

We note that selecting a large learning rate value may cause global minimum overshooting, whereas choosing a small learning rate may necessitate many iterations for the algorithm to converge. Hence, it is reasonable to let the learning rate decrease over time as the algorithm converges to the global minimum. In Figure 2 and Algorithm 1, we present in detail, the framework and the algorithm of the proposed fuzziness reduction model. The proposed learning Algorithm 1: SimFuzzinessReduction keeps adjusting the weight vector θ by moving in the opposite direction to the gradient of the loss function H(θ,ϵ) until it reaches the maximum number of iteration epochs or until the magnitude of the gradient vector becomes below the minimum value ϵ. After convergence, we can feed the new similarity values $\left[g\left(\theta_{0}\times sim\left(D_{0},D_{1}\right),\epsilon \right),g\left(\theta_{0}\times sim\left(D_{0},D_{2}\right),\epsilon \right),\ldots ,g\left(\theta_{0}\times sim\left(D_{n-2},D_{n-1}\right),\epsilon \right)\right]$ to any similarity-based clustering algorithm in order to improve the quality of the produced clustering when the latter is trivial or irrelevant.
**Algorithm 1:** SimFuzzinessReduction
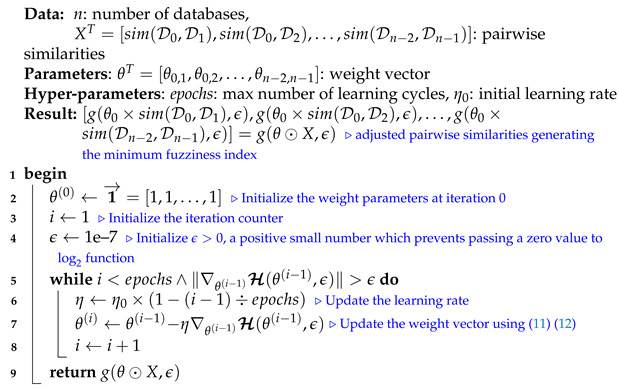


### 3.3. Proposed Coordinate Descent-Based Clustering

In this subsection, we present and discuss our proposed loss function and our coordinate descent-based clustering approach in detail. Unlike the gradient-based clustering in [25,26], our algorithm is learning-rate-free and needs to run at most $(n^{2}-n)/2$ learning cycles to converge to the global minimum, such that *n* is the number of transaction databases. In fact, at each iteration, the largest coordinate variable $\theta_{p,q}$ is selected and popped from a max-heap data structure (initially built by pushing the $(n^{2}-n)/2$ pairwise similarities onto the heap). Then, we minimize our quadratic convex hinge-based loss L(θ) over $\theta_{p,q}$ which is then adjusted by moving in the opposite direction to the gradient of L(θ). This process continues until satisfying a convergence test, which will be defined later in this subsection. Each bloc of selected coordinate variables $\theta_{p,q}$ that have the same value will form a set of edges to be added to our graph G=(D,E). Determining the disjoint connected components in *G* after convergence will allow us to discover the optimal database clusters maximizing the intra-cluster similarity and the inter-cluster distance.

#### 3.3.1. Proposed Loss Function and Algorithm

In order to implement our coordinate descent-based clustering, we propose a quadratic version of the hinge loss L(θ):Rn2→[0,n2−n4], which is a convex function (see proof of Theorem 1) whose minimization problem is formulated as follows:

(14) $$\begin{array}{c} \arg\min_{\theta^{\left(i\right)}}\;L\left(\theta^{\left(i\right)}\right)=\arg\min_{\begin{array}{c} \theta^{\left(i\right)} \end{array}}\;\sum_{r=0}^{n-2}\sum_{s=r+1}^{n-1}\frac{1}{2}\max{\left(0,1-g\left(\theta_{r,s}^{\left(i\right)}\right)\right)}^{2} \end{array}$$

**Figure 3:**
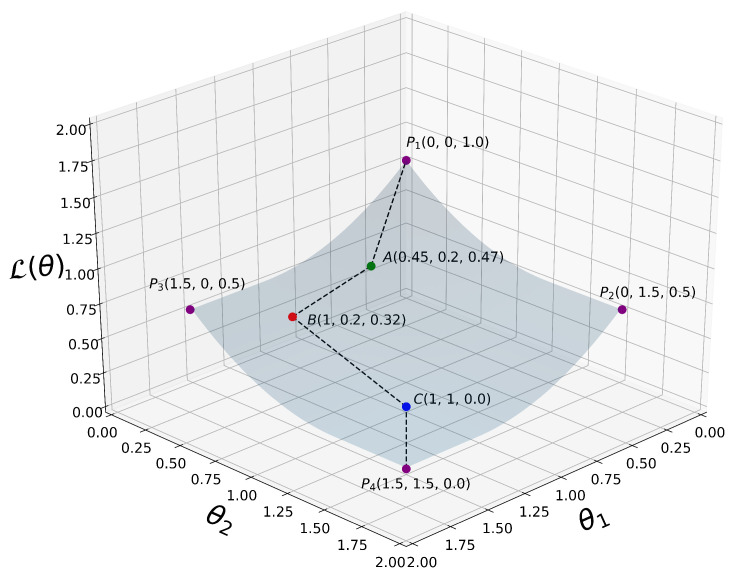
A simplified 3D plot of our proposed loss function L(θ) as defined in (14), where $\theta =[\theta_{1},\theta_{2}]$ for visualization purposes. $P_{1},P_{2},P_{3},P_{4},A,B,C$ are some selected 3D points at which L(θ) is evaluated. From $P_{1}$ all the way down to $P_{4}$, we can clearly see that L(θ) decreases monotonically when the coordinate variables $\theta_{1}$ and $\theta_{2}$ increase their values. That is, $\forall \left(\theta_{1}^{\left(i\right)},\theta_{2}^{\left(i\right)},\theta_{1}^{\left(i-1\right)},\theta_{2}^{\left(i-1\right)}\right)\in R^{4}|\theta_{1}^{\left(i\right)}\geq \theta_{1}^{\left(i-1\right)}\wedge \theta_{2}^{\left(i\right)}\geq \theta_{2}^{\left(i-1\right)},L\left(\theta_{1}^{\left(i\right)},\theta_{2}^{\left(i\right)}\right)\leq L\left(\theta_{1}^{\left(i-1\right)},\theta_{2}^{\left(i-1\right)}\right)$, where *i* is an integer representing the current iteration in our algorithm.

A simplified 3D graph plot of L(θ) is depicted in Figure 3.

Initially, the weight vector $\theta^{T}$ is set to the n2 pairwise similarities $X^{T}=\left[sim\left(D_{0},D_{1}\right),sim\left(D_{0},D_{2}\right),\ldots ,sim\left(D_{n-2},D_{n-1}\right)\right]$, and then each weight component of $\theta^{T}$ is pushed onto a max-heap data structure. At each iteration i=1,…,n2, the weight $\theta_{p,q}^{\left(i\right)}$ (p=0,…,n−2, q=p+1,…,n−1) associated with the current largest similarity value $sim(D_{p},D_{q})$ is popped from the max-heap and is updated as follows:

(15) $$\begin{array}{cc} \theta_{p,q}^{\left(i\right)} & =\theta_{p,q}^{\left(i-1\right)}-\eta \frac{\partial L(\theta^{\left(i-1\right)})}{\partial \theta_{p,q}^{\left(i-1\right)}}\\ \; & =\theta_{p,q}^{\left(i-1\right)}+\eta \left(1-g\left(\theta_{p,q}^{\left(i-1\right)}\right)\right)\frac{\partial g(\theta_{p,q}^{\left(i-1\right)})}{\partial \theta_{p,q}^{\left(i-1\right)}} \end{array}$$

Such that g:R→[0,1] is a differentiable activation function defined as follows:

(16) $$\begin{array}{c} g\left(\theta_{p,q}\right)=\max\left(\theta_{p,q},0\right)-\frac{\mathrm{sgn}(\theta_{p,q}-1)+1}{2}\times \left(\theta_{p,q}-1\right) \end{array}$$

and its partial derivative with respect to the weight $\theta_{p,q}$ is:

(17) $$\begin{array}{c} \frac{\partial g(\theta_{p,q})}{\partial \theta_{p,q}}=\frac{\mathrm{sgn}(\theta_{p,q})+1}{2}-\frac{\mathrm{sgn}(\theta_{p,q}-1)+1}{2} \end{array}$$

We note that sgn:R→{−1,1} is the signum function. The usage of g(·) ensures that each weight $\theta_{p,q}$ is within the range [0,1]. As there is no learning rate and schedule to choose for our coordinate descent-based algorithm, we set η to 1.

**Theorem** **1.** L(θ) (14) *is convex satisfying the following inequality* [46]*:*

(18) L((1−ε)θ(i+1)+εθ(i))≤(1−ε)L(θ(i+1))+εL(θ(i))forallθ(i+1),θ(i)∈Rn2withε∈[0,1]

**Proof.**  To prove the convexity of L(θ), we can show that its Hessian matrix HL is positive semi-definite as follows:

$$\begin{array}{c} H\;L=\frac{\partial^{2}L}{\partial \theta_{p,q}\partial \theta_{r,s}}=\left[\begin{array}{c} \frac{\partial^{2}L\left(\theta \right)}{\partial {\theta^{2}}_{0,1}}=1,\frac{\partial^{2}L\left(\theta \right)}{\partial \theta_{0,1}\partial \theta_{0,2}}=0,\ldots ,\frac{\partial^{2}L\left(\theta \right)}{\partial \theta_{0,1}\partial \theta_{n-2,n-1}}=0\\ \frac{\partial^{2}L\left(\theta \right)}{\partial \theta_{0,2}\partial \theta_{0,1}}=0,\frac{\partial^{2}L\left(\theta \right)}{\partial {\theta^{2}}_{0,2}}=1,\ldots ,\frac{\partial^{2}L\left(\theta \right)}{\partial \theta_{0,2}\partial \theta_{n-2,n-1}}=0\\ \vdots \;\;\;\;\vdots \;\;\ddots \;\;\vdots \\ \frac{\partial^{2}L\left(\theta \right)}{\partial \theta_{n-2,n-1}\partial \theta_{0,1}}=0,\frac{\partial^{2}L\left(\theta \right)}{\partial \theta_{n-2,n-1}\partial \theta_{0,2}}=0,\ldots ,\frac{\partial^{2}L\left(\theta \right)}{\partial^{2}\theta_{n-2,n-1}}=1 \end{array}\right] \end{array}$$

Since H is positive semi-definite satisfying $x^{T}Hx\geq 0$ for all x∈Rn2, L(θ) is convex, and therefore guarantees convergence to the global minimum. □

In order to reach the global minimum of L(θ) (i.e., minL(θ)=0), our learning algorithm needs to set the weight vector θ to $\vec{1}$ (i.e., the unit vector). Consequently, the intra-cluster similarity will reach its maximum value and all the *n* databases will be put into the same cluster resulting in a meaningless partitioning. Therefore, in order to prevent this scenario from occurring, we need to assess the clustering quality after popping all the coordinate variables that have the same weight $\theta_{p,q}$ (i.e., a block of weights having the same value) from the max-heap. This corresponds to generating one candidate clustering by adding the list of edges $\left(D_{p},D_{q}\right)$ satisfying $sim\left(D_{p},D_{q}\right)\geq \theta_{p,q}$ to the graph G=(D,E). Afterward, we need a stopping condition to terminate our algorithm if the current candidate clustering quality is judged to be the optimal one in terms of the similarity-intra cluster $W_{\theta^{\left(i\right)}}\left(D\right)$ and the number of clusters $f_{\theta^{\left(i\right)}}\left(D\right)$. For this purpose, we define the following quasi-convex loss function evaluated at the *i*-th iteration:

(19) $$\begin{array}{cc} L(\theta^{\left(i\right)}) & =\frac{1}{2}{\left(f_{\theta^{\left(i\right)}}\left(D\right)-W_{\theta^{\left(i\right)}}\left(D\right)\right)}^{2}\\ \; & =\frac{1}{2}{\left(f_{\theta^{\left(i\right)}}\left(D\right)-\varphi \left({\theta^{\left(i\right)}}^{T}\right)\cdot X\right)}^{2}\\ \; & =\frac{1}{2}{\left(f_{\theta^{\left(i\right)}}\left(D\right)-\sum_{p=0}^{n-2}\sum_{q=p+1}^{n-1}\;sim\left(D_{p},D_{q}\right)\times \varphi \left(\theta_{p,q}^{\left(i\right)}\right)\right)}^{2} \end{array}$$

where φ:Rn2→{0,1}n2, $\varphi \left(\theta \right)=\frac{\mathrm{sgn}(\theta -1)+1}{2}$.

Our algorithm terminates right after it reaches the global minimum of L(·). In other words, if $L\left(\theta^{\left(i\right)}\right)\leq L\left(\theta^{\left(i-1\right)}\right)$, then we continue updating the weight vector, the clustering labels and save the optimal partitioning found so far. Otherwise, the algorithm terminates as it has reached the global minimum $L(\theta^{\left(i-1\right)})$, and therefore, the optimal partitioning saved so far is returned as the ideal clustering of the *n* transactional databases. This stopping condition is only possible due to the quasi-convexity of L(·).

**Theorem** **2.** L(θ) (19) *is quasi-convex satisfying the following inequality [46]:*

(20) L((1−ε)θ(i+1)+εθ(i))≤max{L(θ(i+1)),L(θ(i))}forallθ(i+1),θ(i)∈Rn2withε∈[0,1]

**Proof.**  To prove the quasi-convexity of L(θ), we need to demonstrate the validity of (20). First, since $f_{\theta }\left(D\right)$ is a decreasing function on the range [0,1], it is then both quasi-concave and quasi-convex satisfying the following: $f\left(\left(1-\varepsilon \right)\theta^{\left(i+1\right)}+\varepsilon \theta^{\left(i\right)}\right)\leq \max\left\{f\left(\theta^{\left(i+1\right)}\right),f\left(\theta^{\left(i\right)}\right)\right\}$ for all θ(i+1),θ(i)∈Rn2 with ε∈[0,1]. Since $W_{\theta }\left(D\right)$ is an increasing function on the range [0,1], it is then both quasi-concave and quasi-convex satisfying the following: $W\left(\left(1-\varepsilon \right)\theta^{\left(i+1\right)}+\varepsilon \theta^{\left(i\right)}\right)\geq \min\left\{W\left(\theta^{\left(i+1\right)}\right),W\left(\theta^{\left(i\right)}\right)\right\}$ for all θ(i+1),θ(i)∈Rn2 with ε∈[0,1]. By subtracting the two last inequalities, we obtain: $\left(f\left(\left(1-\varepsilon \right)\theta^{\left(i+1\right)}+\varepsilon \theta^{\left(i\right)}\right)-W\left(\left(1-\varepsilon \right)\theta^{\left(i+1\right)}\right)+\varepsilon \theta^{\left(i\right)}\right)\leq \left(\max\left\{f\left(\theta^{\left(i+1\right)}\right),f\left(\theta^{\left(i\right)}\right)\right\}-\min\left\{W\left(\theta^{\left(i+1\right)}\right),W\left(\theta^{\left(i\right)}\right)\right\}\right)$. Since $f\left(\theta^{\left(i+1\right)}\right)\leq f\left(\theta^{\left(i\right)}\right)$ and $W\left(\theta^{\left(i+1\right)}\right)\geq W\left(\theta^{\left(i\right)}\right)$, the right side of the resulting inequality is equal to $f\left(\theta^{\left(i\right)}\right)-W\left(\theta^{\left(i\right)}\right)$, which could be set equal to $\max\{f\left(\theta^{\left(i+1\right)}\right)-W\left(\theta^{\left(i+1\right)}\right),f\left(\theta^{\left(i\right)}\right)-W\left(\theta^{\left(i\right)}\right)\}$. Finally, by squaring and dividing both sides of the inequality by 2, we get a variation on the Jensen inequality for quasi-convex functions [46] as defined in (20). Hence, L(θ) is quasi-convex. □

#### 3.3.2. Time Complexity Analysis

**Figure 4:**
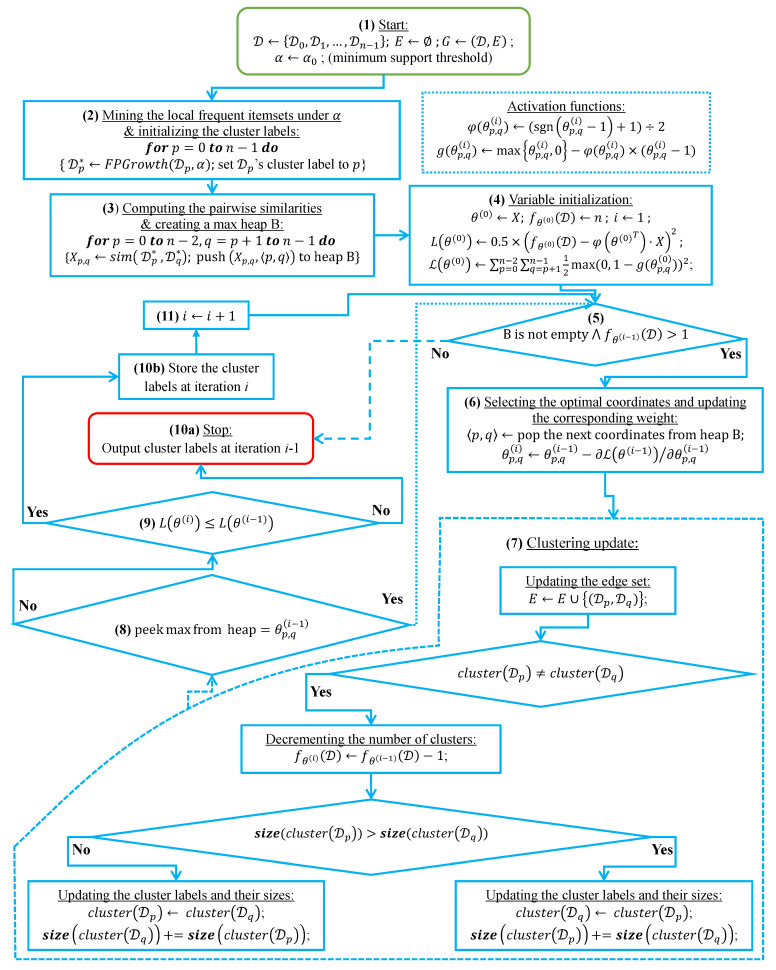
The coordinate descent-based clustering model depicted in eleven steps.

In this subsection, we analyze the time complexity of our coordinate descent-based clustering algorithm presented in Algorithm 2, named CDClustering, which depends on the two subroutines presented in Algorithm 3: union and Algorithm 4: cluster. We note that the superscript *i* enclosed in round brackets, i.e., $\theta_{p,q}^{\left(i\right)}$, is used to indicate the iteration number at which a given variable $\theta_{p,q}$ has been assigned a value. The proposed algorithm takes as argument the n2 pairwise similarities $X^{T}=\left[sim\left(D_{0},D_{1}\right),sim\left(D_{0},D_{2}\right),\ldots ,sim\left(D_{n-2},D_{n-1}\right)\right]$ and outputs the optimal clustering minimizing our proposed loss function L(θ) (14). First, the weight vector $\theta^{T}$ is initially set equal to $X^{T}$. Afterward, coordinate descent and back-propagation are used to search for the optimal weight vector $\theta^{T}$, which minimizes our hinge-based objective L(θ). Through each learning cycle *i*, one coordinate variable $\theta_{p,q}$ is popped from a max-heap. Then, $\theta_{p,q}$ is updated by making the optimal step in the opposite direction to the gradient of L(θ). The weights $\theta_{p,q}$ (p=0,…,n−2,q=p+1,…,n−1) attaining the maximum value of 1 will have their corresponding database pairs $\left(D_{p},D_{p}\right)$ put into the same cluster. By using a max-heap data structure within our coordinate descent algorithm, we optimally choose the current largest variable $\theta_{p,q}^{\left(i\right)}$ at each iteration *i* such that taking the partial derivative of our loss L(θ) with respect to $\theta_{p,q}$ allows us to attain the next steepest descent minimizing L(θ) without using a learning rate. This way, the maximum number of iterations required for our algorithm to converge is less than or equal to $(n^{2}-n)/2$, i.e., the number of the pairwise similarities. Initially, the number of clusters $f_{\theta }\left(D\right)$ is set equal to the number of transactional databases *n*. Then, in order to keep track of the database clusters, their number $f_{\theta }\left(D\right)$ and their sizes, we implement a *disjoint-set* data structure [47], which consists of an array A[0,…,n−1] of *n* integers managed by two main operations: *cluster* and *union*. Each cluster $C_{p}$ is represented by a tree whose root index *p* satisfies A[p]=−1, and a database $D_{q}$ belonging to the cluster $C_{p}$ satisfies A[q]=p. Therefore, the *cluster* function is called recursively to find the label assigned to the database index *p* (passed in as argument) by moving down the tree towards the root (i.e., A[p]=−1). On the other hand, the *union* procedure links two disjoint clusters $C_{p}$ and $C_{q}$ by making the root of the smaller tree point to the root of the larger one in A[0,…,n−1]. The algorithms corresponding to *union* and *cluster* are presented in Algorithm 3 and Algorithm 4, respectively. Let s=n2 be the size of the weight vector $\theta^{T}$. The time complexity of building the max-heap is O(s) and the time complexity of the proposed Algorithm 2: CDClustering is $O(s+h\log_{2}\left(n\right))$, such that h∈[1,s] is the number of learning cycles run until global minimum convergence, and $O(\log_{2}\left(n\right))$ is the time complexity of one pop operation from the heap. The proposed model is also illustrated in Figure 4. Since it is meaningless to return a single cluster consisting of all the *n* databases, if the clustering obtained at step (10a) is trivial (i.e., all the *n* databases are put together in one class or each single database stands alone in its own cluster), then we first need to run the model proposed in Figure 2 on the pairwise similarities to reduce the associated intrinsic fuzziness measured in (9). Afterward, we can apply the proposed model Figure 4 on the new adjusted similarity values to obtain more relevant results.
**Algorithm 2:** CDClustering
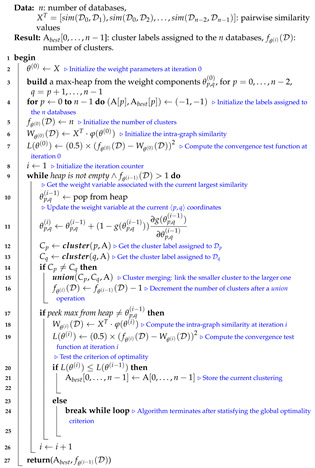

**Algorithm 3:** ***union***
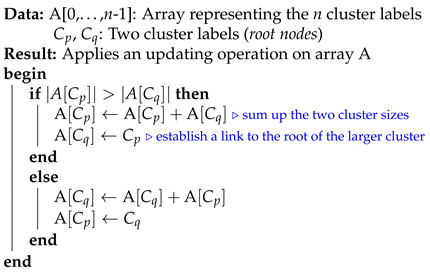

**Algorithm 4:** ***cluster***
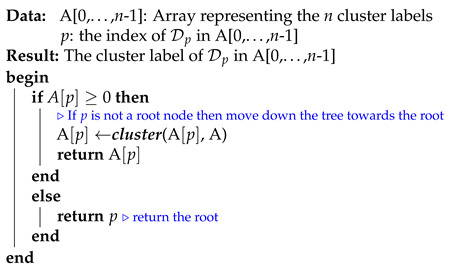


## 4. Performance Evaluation

To assess the performance of the proposed clustering algorithm, we carried out numerous experiments on real and synthetic datasets, including Zoo [48], Iris [48], Mushroom [48] and T10I4D100K [49]. To simulate a multi-database environment, we have partitioned each dataset horizontally into *n* partitions $D_{1},D_{2},\ldots ,D_{n}$, such that n∈{12,10,6,4}. Afterward, given a minimum support threshold α∈{0.5,0.2,0.03}, we run FP-Growth [1] on each partition $D_{i}$ (i=1,…n) to discover the local frequent itemsets (FIs) corresponding to each partition. All the details related to the partition sizes and their corresponding FIs are shown in Table A1. We note that the fifth column of Table A1 reports the number of FIs discovered in the entire dataset, whereas the most right column of the same table reports the number of FIs aggregated from the local FIs mined from the partitions in each cluster.

**Figure A1:**
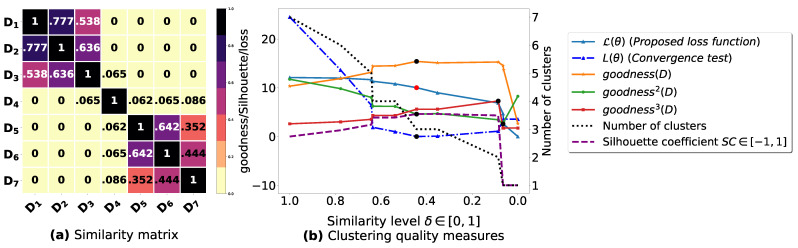
(**a**): represents a similarity matrix between 7 databases from [25]. We note that (a) is built by calling sim (3) on the frequent itemsets (FIs) mined from $D_{p}$ (p=1,…,7) under a threshold α=0.42. (**b**): represents the graph plots corresponding to ${\mathrm{goodness}}^{3}\left(D\right)\times 10$ [21], ${\mathrm{goodness}}^{2}\left(D\right)\times 10$ [23], goodness(D) [20], the silhouette coefficient SC×10 [43], our convergence test function L(θ), our proposed loss function L(θ)×5 and the number of generated clusters.

**Figure A2:**
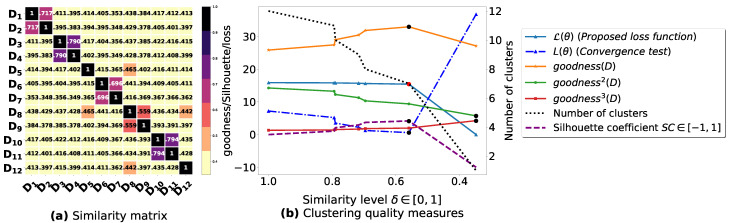
(**a**): represents a similarity matrix between 12 databases partitioned from Zoo dataset [48]. We note that (**a**) is built by calling sim (3) on the frequent itemsets (FIs) mined from $D_{p}$ (p=1,…,12) under a threshold α=0.5. (**b**): represents the graph plots corresponding to ${\mathrm{goodness}}^{3}\left(D\right)\times 10$ [21], ${\mathrm{goodness}}^{2}\left(D\right)\times 10$ [23], goodness(D) [20], the silhouette coefficient SC×10 [43], our convergence test function L(θ)÷10, our proposed loss function L(θ)×2 and the number of generated clusters.

**Figure A3:**
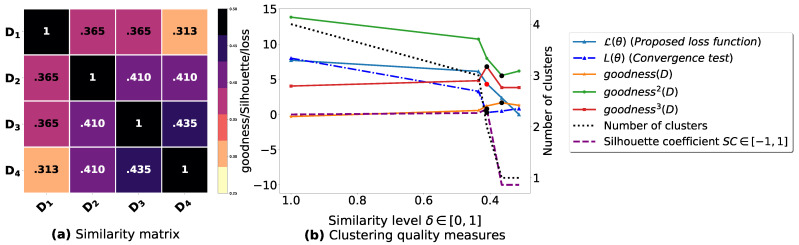
(**a**): represents a similarity matrix between 4 databases partitioned from Mushroom dataset [48] without applying the fuzziness reduction model in Figure 2. We note that (a) is built by calling sim (3) on the frequent itemsets (FIs) mined from $D_{p}$ (p=1,…,4) under a threshold α=0.5. (**b**): represents the graph plots corresponding to ${\mathrm{goodness}}^{3}\left(D\right)\times 10$ [21], ${\mathrm{goodness}}^{2}\left(D\right)\times 10$ [23], goodness(D) [20], the silhouette coefficient SC×10 [43], our convergence test function L(θ), our proposed loss function L(θ)×10 and the number of generated clusters.

**Figure A4:**
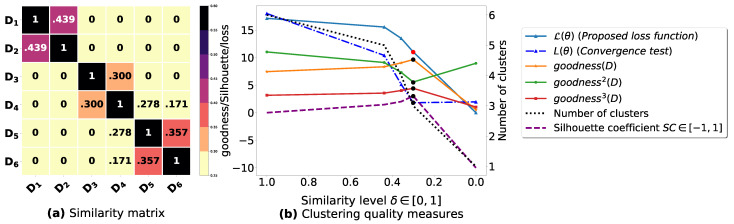
(**a**): represents a similarity matrix between 6 databases partitioned from Iris dataset [48]. We note that (a) is built by calling sim (3) on the frequent itemsets (FIs) mined from $D_{p}$ (p=1,…,6) under a threshold α=0.2. (**b**): represents the graph plots corresponding to ${\mathrm{goodness}}^{3}\left(D\right)\times 10$ [21], ${\mathrm{goodness}}^{2}\left(D\right)\times 10$ [23], goodness(D) [20], the silhouette coefficient SC×10 [43], our convergence test function L(θ), our proposed loss function L(θ)×10 and the number of generated clusters.

**Figure A5:**
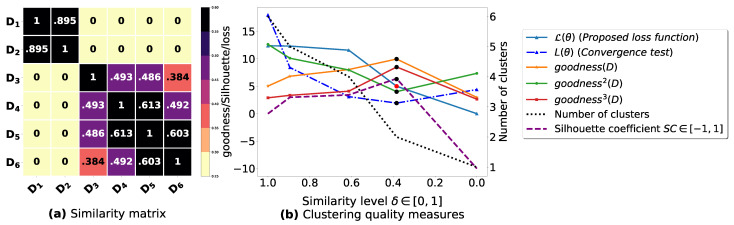
(**a**): represents a similarity matrix between 6 databases including 4 databases partitioned from the real dataset Mushroom [48] and 2 databases partitioned from the real dataset Zoo [48]. We note that (**a**) is built by calling sim (3) on the frequent itemsets (FIs) mined from $D_{p}$ (p=1,…,6) under a threshold α=0.5. (**b**): represents the graph plots corresponding to ${\mathrm{goodness}}^{3}\left(D\right)\times 10$ [21], ${\mathrm{goodness}}^{2}\left(D\right)\times 10$ [23], goodness(D) [20], the silhouette coefficient SC×10 [43], our convergence test function L(θ), our proposed loss function L(θ)×10 and the number of generated clusters.

**Figure A6:**
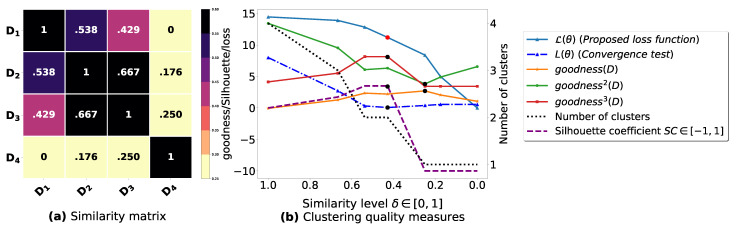
(**a**): represents a similarity matrix between 4 databases from [39]. (**b**): represents the graph plots corresponding to ${\mathrm{goodness}}^{3}\left(D\right)\times 10$ [21], ${\mathrm{goodness}}^{2}\left(D\right)\times 10$ [23], goodness(D) [20], the silhouette coefficient SC×10 [43], our convergence test function L(θ), our proposed loss function L(θ)×10 and the number of generated clusters.

**Figure A7:**
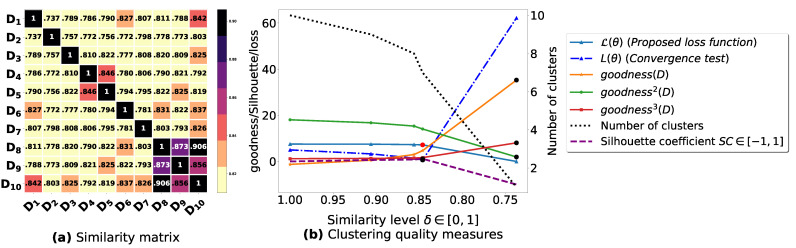
(**a**): represents a similarity matrix between 10 databases partitioned from T10I4D100K [49]. We note that (a) is built by calling sim (3) on the frequent itemsets (FIs) mined from $D_{p}$ (p=1,…,10) under a threshold α=0.03. (**b**): represents the graph plots corresponding to ${\mathrm{goodness}}^{3}\left(D\right)\times 10$ [21], ${\mathrm{goodness}}^{2}\left(D\right)\times 10$ [23], goodness(D) [20], the silhouette coefficient SC×10 [43], our convergence test function L(θ)÷10, our proposed loss function L(θ)×10 and the number of generated clusters.

The proposed similarity measure sim (3) is called on the $(n^{2}-n)/2$ pairs of FIs to compute the n×n similarity matrices shown in Figure A1a, Figure A2a, Figure A3a, Figure A4a, Figure A5a, Figure A6a, and Figure A7a. Next, using the obtained pairwise similarities, candidate clusterings are produced via the process described in Section 3.1.2, and then evaluated using the clustering quality measures defined in Table 3, including SC(D) [43], $goodness^{3}\left(D\right)$ [21], $goodness^{2}\left(D\right)$ [23], goodness(D) [20] and our proposed loss function L(θ) (14). The graphs corresponding to the studied goodness measures are shown in Figure A1b, Figure A2b, Figure A3b, Figure A4b, Figure A5b, Figure A6b, and Figure A7b, where the optimal point (maximum or minimum) of each objective function is depicted as a black dot on its corresponding graph, except that for the graph of our loss function L(θ), there is a red dot representing the value $L(\arg\min_{\begin{array}{c} \theta \end{array}}L\left(\theta \right))$ (i.e., the optimal point at which our algorithm terminates). It is worth mentioning that due to scale differences, we sometimes multiply or divide our loss function L(θ), $goodness^{3}\left(D\right)$ [21] and $goodness^{2}\left(D\right)$ [23] by a scaling number to stretch or shrink their graphs in the direction of the *y*-axis. The experimental results depicted in Figure A1, Figure A2, Figure A3, Figure A4, Figure A5, Figure A6, and Figure A7 are summarized in Table A2, such that δ∈[0,1] is the ideal similarity threshold for which a goodness measure attains its optimal point. Python version 3.9.2 was used to implement all the algorithms, and the codes were run on a Ubuntu-20.04 server equipped with an Intel(R) Xeon(R) CPU clocked at 2.30 GHz with 50 GB available Disk capacity and 12 GB of available RAM.

### 4.1. Similarity Accuracy Analysis

To demonstrate the efficiency of sim (3), let us have three transaction databases, $|D_{1}|$ = 200, $|D_{2}|$ = 300 and $|D_{3}|$ = 200 with their corresponding local frequent itemsets: $FIS(D_{1},0.2)$ = {〈C,0.2〉,〈B,0.2〉,〈A,0.2〉}, $FIS(D_{2},0.15)$ = {〈E,0.9〉,〈C,0.2〉,〈B,0.2〉,〈A,0.2〉} and $FIS(D_{3},0.25)$ = {〈E,0.9〉} mined at different minimum support threshold values $\alpha_{1}=0.2$, $\alpha_{2}=0.15$ and $\alpha_{3}=0.25$, respectively. Now, clustering the three databases using the algorithm BestDatabaseClustering [22] equipped with two different similarity measures, simi proposed in [20] and our proposed similarity measure sim (3), shows the results reported in Table 4. We note that goodness [20] is a clustering quality measure, such that the higher the value of goodness for a given candidate clustering C, the better the quality of C.

From Table 4, we notice that using our similarity measure sim (3), we have obtained a larger intra-cluster similarity, a larger inter-cluster distance and a larger goodness [20]. Now, let us synthesize the global frequent itemsets from the clusters containing more than one database, i.e., $C_{2,3}=\left\{D_{2},D_{3}\right\}$ and $C_{1,2}=\left\{D_{1},D_{2}\right\}$. The obtained results are shown in Table 5, such that $\alpha_{2,3}=\frac{300\times 0.15+200\times 0.25}{300+200}=0.19$ and $\alpha_{1,2}=\frac{200\times 0.2+300\times 0.15}{200+300}=0.17$ are the minimum support thresholds corresponding to $C_{2,3}$ and $C_{2,3}$ respectively. As we can see, the similarity measure simi [20] captures only high frequency itemsets (supp≈1), such as *E*, and neglects low support frequent itemsets (i.e., whose supports are immediately above the minimum threshold α with supp∈[α,α+ϵ] and ϵ is a very small number), such as *A*, *B* and *C*. This characteristic gives a high similarity value to database pairs sharing only one or very few high frequency itemsets. On the other hand, database pairs sharing many frequent itemsets with a low support will be assigned a lower similarity. However, once the clustering is done, we will be interested in the patterns discovered from each cluster individually, such as the *high-vote patterns* [32] and the *exceptional patterns* [31]. That is why our similarity measure estimates the patterns post-mined from each cluster $C_{p,q}=\left\{D_{p},D_{q}\right\}$ in order to compute $sim(D_{p},D_{q})$. Since our similarity measure focuses on maximizing the number of frequent itemsets synthesized from each cluster $C_{p,q}\subseteq D$, only relevant clusters will be assigned a large similarity value.

### 4.2. Fuzziness Reduction Analysis

**Figure 5:**
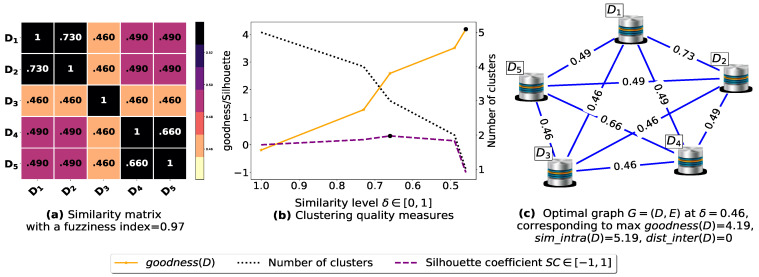
(**a**) A 5 × 5 similarity matrix between five transactional databases before applying our fuzziness reduction model. (**b**) Represents the graph plots corresponding to goodness(D) [20], the silhouette coefficient [43] and the number of clusters. (**c**) Represents the optimal graph obtained at maxgoodness(D) [20].

**Figure 6:**
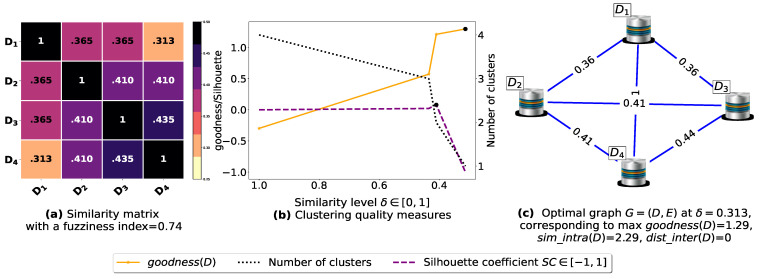
(**a**) Represents a similarity matrix between four databases partitioned from the Mushroom dataset [48]. We note that (**a**) is built by calling *sim* (3) on the frequent itemsets (FIs) mined from *D_p_* (*p* = 1, …, 4) under a threshold *α* = 0.5. (**b**) Represents the graph plots corresponding to goodness(D) [20], the silhouette coefficient [43] and the number of clusters. (**c**) Represents the optimal graph obtained at maxgoodness(D) [20].

To demonstrate the importance of reducing the fuzziness associated with a similarity matrix, we run the clustering algorithm BestDatabaseClustering [22] on two similarity matrices in Figure 5a and Figure 6a. The obtained results in terms of the optimal clustering, maxgoodness(D) [20], the optimal similarity level $\delta_{opt}$ (i.e., the similarity level at maxgoodness(D)) and the silhouette coefficient SC(D) [43] at $\delta_{opt}$ are shown in Figure 5b,c and Figure 6b,c corresponding to rows 1 and 2 of Table 6, respectively. From the obtained results, we can clearly see that when the similarity matrices are centered around the mean value 0.5, the fuzziness index becomes larger and closer to 1, and BestDatabaseClustering [22] could not return a meaningful clustering since it has put all the *n* databases into the same cluster.

**Figure 7:**
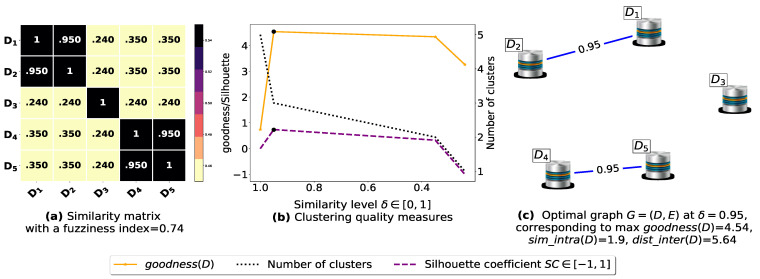
(**a**) A 5 × 5 similarity matrix obtained after applying our fuzziness reduction model on Figure 5a. (**b**) Represents the graph plots corresponding to goodness(D) [20], the silhouette coefficient [43] and the number of clusters. (**c**) Represents the optimal graph obtained at maxgoodness(D) [20].

**Figure 8:**
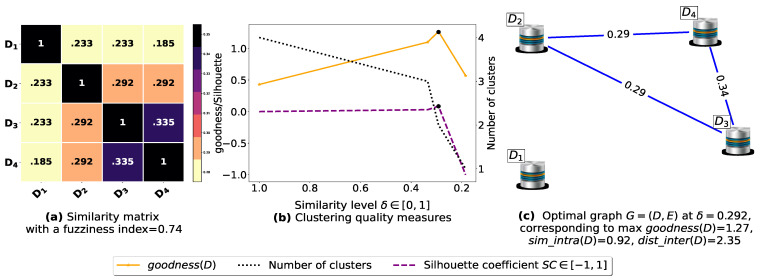
(**a**) Represents the similarity table generated after applying our fuzziness reduction model on Figure 6a. (**b**) Represents the graph plots corresponding to goodness(D) [20], the silhouette coefficient [43] and the number of clusters. (**c**) Represents the optimal graph obtained at maxgoodness(D) [20].

Now, let us run our fuzziness reduction model on the previous similarity matrices and depict the adjusted similarity matrices in Figure 7a and Figure 8a, respectively. Afterward, we run BestDatabaseClustering [22] on the new similarity matrices and show the clustering results in Figure 7b,c and Figure 8b,c corresponding to rows 3 and 4 of Table 6, respectively. As we can see, after reducing the fuzziness index associated with the previous similarity matrices in Figure 5a and Figure 6a, the algorithm BestDatabaseClustering [22] was able to produce meaningful non-trivial clusterings with an increase in the silhouette coefficient SC(D) [43] for both similarity matrices in Figure 7a and Figure 8a.

### 4.3. Convexity and Clustering Analysis

In this part of our experiments, we analyze the convex behavior of the proposed clustering quality functions L(θ) (19) and L(θ) (14), and we also examine the non-convexity of the existing goodness measures in [20,21,23,43]. Additionally, we compare the clustering produced by our algorithm and the ones generated at the optimal points of the previous compared goodness measures (i.e., at maxgoodness(D) [20], $\min goodness^{2}\left(D\right)$ [23] and $\max goodness^{3}\left(D\right)$ [21]) with the underlying ground-truth cluster labels. When the actual clustering is unknown, we replace it with the partitioning obtained at the maximum value of the silhouette coefficient [43], that is, at maxSC(D). All the graphs corresponding to our loss functions and the compared goodness measures in Table 3 are plotted in Figure A1b, Figure A2b, Figure A3b, Figure A4b, Figure A5b, Figure A6b, and Figure A7b, where the *x*-axis represents the similarity levels δ at which multiple candidate clusterings are generated and evaluated.

Consider the 7×7 similarity matrix shown in Figure A1a. From the graphs plotted in Figure A1b and according to the results shown in the first row of Table A2, we can see that using our loss function L(θ) and goodness(D) [20], we were able to find the optimal clustering $\left\{C_{1}=\left\{D_{3},D_{2},D_{1}\right\},C_{2}=\left\{D_{4}\right\},C_{3}=\left\{D_{7},D_{6},D_{5}\right\}\right\}$ at a similarity level δ=0.44 where the silhouette coefficient reaches its maximum value SC(D)=0.46. On the other hand, $goodness^{3}\left(D\right)$ [21] and $goodness^{2}\left(D\right)$ [23] did not successfully discover the partitioning maximizing the silhouette coefficient. Additionally, we observe that the proposed convergence test function L(θ) has a quasi-convex behavior (see proof of Theorem 2). This allows us to terminate the clustering process right after reaching the global minimum. Conversely, the graphs corresponding to $goodness^{2}\left(D\right)$ [23] and goodness(D) [20] have local optima. Consequently, it is required to explore about $(n^{2}-n)/2$ similarity levels in order to generate and evaluate all the candidate clusterings possible.

Now, let us examine the results of some experiments that we have conducted on the synthetic and real-world datasets shown in Table A1. From Figure A2b and Figure A7b (the last and second rows of Table A2), we observe that $goodness^{3}\left(D\right)$ [21] and $goodness^{2}\left(D\right)$ [23] attain their optimal values when all the partition databases are clustered together in one class. The same phenomenon is observed in Figure A3b, Figure A6b and Figure A7b (the last, the sixth and the third rows of Table A2), where both $goodness^{2}\left(D\right)$ [23] and goodness(D) [20] have put all the databases into one cluster.

In contrast, the proposed loss function L(θ) has successfully identified the clustering for which the silhouette coefficient SC is maximum. Precisely, in Figure A7b, which corresponds to the last row of Table A2), L(θ) was the only clustering quality measure which has properly identified the ideal 7-class clustering at δ=0.846.

From the obtained graphs in Figure A1, Figure A2, Figure A3, Figure A4, Figure A5, Figure A6, and Figure A7, we notice that $goodness^{3}\left(D\right)$ [21], $goodness^{2}\left(D\right)$ [23] and goodness(D) [20] are neither quasi-concave nor quasi-convex on the domain [0,1]. As a result, we observe the existence of local optimum points on their corresponding graphs, which makes the search of the global optimum a difficult problem to solve without exploring all the local solutions.

Conversely, we observe that our loss function L(θ) (14) is monotonically decreasing all the time and $L(\hat{\theta })=0$ at $\hat{\theta }=\arg\min_{\begin{array}{c} \theta \end{array}}L\left(\theta \right)=\vec{1}$. This corresponds to the similarity level δ=0 where all the *n* databases are put into the same single cluster. To avoid this case from occurring, we used the quasi-convex function L(θ) (19) as a convergence test function to terminate our algorithm at the point $L(\arg\min_{\begin{array}{c} \theta \end{array}}L\left(\theta \right))$ corresponding to the red dot on the graph of our loss function L(θ). Moreover, it is worth noting that for every two real n2-dimensional vectors $\theta^{\left(i\right)}$ and $\;\theta^{\left(i+1\right)}$, where $L\left(\theta^{\left(i+1\right)}\right)\leq L\left(\theta^{\left(i\right)}\right)$, the line that joins the points ($\theta^{\left(i+1\right)},L\left(\theta^{\left(i\right)}\right)$) and ($\theta^{\left(i\right)},L\left(\theta^{\left(i\right)}\right)$) remains above L(θ), which is observed in Figure A1, Figure A2, Figure A3, Figure A4, Figure A5, Figure A6, and Figure A7. Therefore, using the proposed loss function L(θ) (14) along with L(θ) (19) guarantees global minimum convergence.

In the fifth and the most right columns of Table A1, we compare the number of frequent itemsets (FIs) mined from all the partitions of a given dataset D with the FIs mined from each single cluster $C_{j}$ consisting of similar partitions from the same dataset, where $\cap_{j=1}^{k}\left\{C_{j}\right\}=\emptyset$ and $\cup_{j=1}^{k}\left\{C_{j}\right\}=D$. We notice that mining all the partitions from datasets Iris [48] and Zoo [48] did not result in discovering any valid frequent itemset. Whereas, mining each individual cluster of partitions from the datasets Iris and Zoo has led to the discovery of new patterns in each cluster $C_{j}$.

In Table A3, we report the similarity levels $\delta_{opt}$ at which the clustering evaluation measures goodness(D) [20], $goodness^{2}\left(D\right)$ [23], $goodness^{3}\left(D\right)$ [21], the silhouette coefficient SC [43] and our proposed loss function L(θ) attain their optimal values in Figure A1, Figure A2, Figure A3, Figure A4, Figure A5, Figure A6, and Figure A7. We note that, the fraction $\frac{\left|\{\delta_{1},\ldots ,\delta_{stop}\}\right|}{\left|\{\delta_{1},\ldots ,\delta_{m}\}\right|}$ in Table A3 represents the number of similarity levels required to test the convergence and terminate divided by the number of all similarity levels *m*. We note that opt is the index of the optimal similarity level according to a given clustering quality measure. Since our proposed algorithm is based on a convex loss function, we notice that stop=opt<m. On the other hand, as for the compared algorithms, which are based on non-convex objectives, we notice that stop=m. Therefore, our algorithm requires the least number of similarity levels (opt out of *m*) in order to converge and terminate, which makes our algorithm faster than the compared algorithms in [21,22,23], requiring to generate and evaluate all the *m* candidate clusterings in order to return the optimal one.

All the previous results confirm that using our loss function L(θ) (14) along with L(θ) (19), we have identified the ideal clustering for which the silhouette coefficient SC [43] is maximum and we have also improved the quality of the frequent itemsets (FIs) mined from the multiple databases partitioned from the datasets in Table A1.

### 4.4. Clustering Error and Running Time Analysis

In this experimental part, we compare the running time of the proposed clustering algorithm with the execution times of two clustering algorithms for multi-database mining (MDM), namely GDMDBClustering [25] and BestDatabaseClustering [22], all run on the same random data samples. We also calculate how the clusterings produced by our algorithm and the compared models are different from the ground-truth clustering. For this purpose, we propose an error function in (21), which measures the difference between two given clusterings P and Q.

First, we generated *n* = 30 to *N* = 120 isotropic Gaussian blobs using the scikit-learn generator [50], such that the number of features for each *n* blobs is set to random.randint(2,10), while the number of clusters is set to $\left\lfloor \frac{n}{2}\right\rfloor$. In Table 7, we present a brief summary of the random blobs generated via scikit-learn [50].

**Figure A8:**
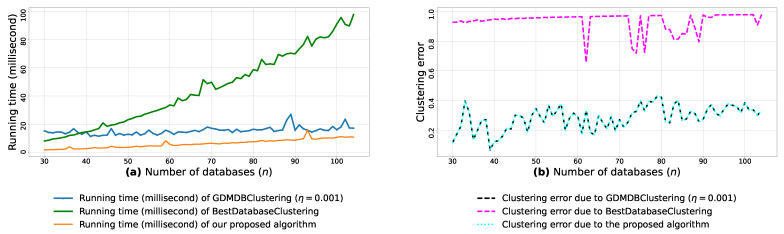
(**a**): represents the running times corresponding to GDMDBClustering [25] (with a learning rate η=0.001), BestDatabaseClustering [22] and the proposed algorithm obtained when executed on n=30,…,120 isotropic Gaussian blobs generated using scikit-learn generator [50]. (**b**): represents the clustering error graphs (21) due to GDMDBClustering [25], BestDatabaseClustering [22] and the proposed algorithm.

**Figure A9:**
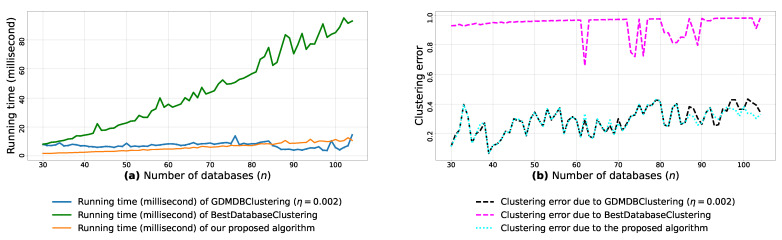
(**a**): represents the running times corresponding to GDMDBClustering [25] (with a learning rate η=0.002), BestDatabaseClustering [22] and the proposed algorithm obtained when executed on n=30,…,120 isotropic Gaussian blobs generated using scikit-learn generator [50]. (**b**): represents the clustering error graphs (21) due to GDMDBClustering [25], BestDatabaseClustering [22] and the proposed algorithm.

**Figure A10:**
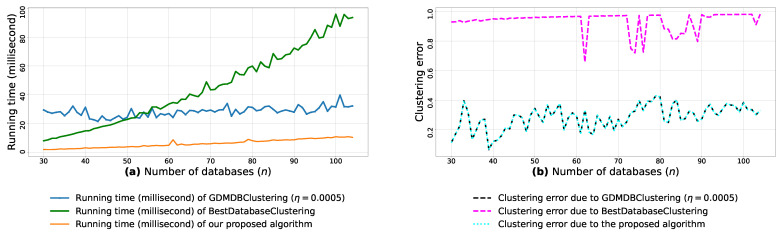
(**a**): represents the running times corresponding to GDMDBClustering [25] (with a learning rate η=0.0005), BestDatabaseClustering [22] and the proposed algorithm obtained when executed on n=30,…,120 isotropic Gaussian blobs generated using scikit-learn generator [50]. (**b**): represents the clustering error graphs (21) due to GDMDBClustering [25], BestDatabaseClustering [22] and the proposed algorithm.

Afterward, we use the *min-max* scaling [51] to normalize each feature (out of the *m* features) into the interval [0,1]. Then, for each *n* blobs, every pair of *m*-dimensional blobs is passed as arguments to the function sim (3) in order to compute the $(n^{2}-n)/2$ pairwise similarities between the *n* blobs. We then run the proposed algorithm, GDMDBClustering [25] (with three different learning rate values) and BestDatabaseClustering [22] on each of the $(n^{2}-n)/2$ pairwise similarities (n=30,…,120) and plot their running time graphs in Figure A8a, Figure A9a and Figure A10a, and then plot the clustering error graphs in Figure A8b, Figure A9b and Figure A10b.

Without loss of generality, assume Q is the ground-truth clustering (i.e., the actual clusters) of the current *n* blobs $D=\{D_{1},D_{2},\ldots ,D_{n}\}$ generated via scikit-learn [50], and assume P is the partitioning of D produced by a given clustering algorithm. To measure how far is P from Q, we propose the error function $E_{n}\left(P,Q\right)\in \left[0,1\right]$ to be defined as follows:

(21) $$E_{n}\left(P,Q\right)=\frac{|Pairs_{Q}\backslash Pairs_{P}|+|Pairs_{P}\backslash Pairs_{Q}|}{|Pairs_{Q}|+|Pairs_{P}|}$$

where $|Pairs_{P}|$ is the number of all the database pairs obtained from every cluster in P and $|Pairs_{P}\backslash Pairs_{Q}|$ is the number of all the database pairs that only exist in $Pairs_{P}$ and that cannot be found in $Pairs_{Q}$. We note that $E_{n}\left(P,Q\right)$ approaches the maximum value of 1 (i.e., $E_{n}\left(P,Q\right)\approx 1$) when P and Q are too different and do not share many database pairs in common (i.e., $|Pairs_{P}\cap Pairs_{Q}|\approx 0$). Conversely, $E_{n}\left(P,Q\right)\approx 0$ when the clustering P and Q are too similar, i.e., they share the maximum number of pairs $\left(D_{p},D_{q}\right)$.

We also define the average of the N−n+1 clustering errors, which could also be seen as the mean absolute clustering error:

(22) $$\bar{E(P,Q)}=\frac{\sum_{n}^{N}E_{n}\left(P,Q\right)}{N-n+1}$$

From the obtained results in Figure A8a, Figure A9a and Figure A10a, we observe a rapid increase in the running time of BestDatabaseClustering [22] as the number of generated blobs (*n*) increases linearly. This is due to the fact that BestDatabaseClustering needs to generate and evaluate approximately $(n^{2}-n)/2$ candidate clusterings in order to find the optimal clustering for which the non-convex function goodness(D) [20] is maximum. In fact, goodness(D) suffers from the existence of local maxima, which requires exploring all the local candidate solutions in order to find the global maximum. On the other hand, using the proposed convex loss function L(θ) and the quasi-convex convergence test function L(θ) allows us to stop the clustering process at $L(\arg\min_{\begin{array}{c} \theta \end{array}}L\left(\theta \right))$. Consequently, this avoids generating unnecessary candidate clusterings, and hence reduces the CPU overhead. Since our algorithm is independent of the learning rate η, the running time of our algorithm is the same in all Figure A8a, Figure A9a and Figure A10a. Whereas, the running time of GDMDBClustering [25] increases for smaller learning rate values (e.g., Figure A10) and decreases when we set larger learning rate values (e.g., Figure A9), but this comes at the cost of having an increased clustering error.

Next, by examining the three clustering error graphs in Figure A8b, Figure A9b and Figure A10b, we observe that BestDatabaseClustering [22] has the largest clustering error among the three algorithms with a clustering average error $\bar{E(P,Q)}=0.936$. In fact, on average, BestDatabaseClustering [22] tends to group all the current *n* blobs (n=30,…,120) in one single cluster. On the other hand, our proposed algorithm and GDMDBClustering [25] produce clusterings that are close to the ground-truth clustering predetermined by the scikit-learn generator [50]. In fact, the average clustering error due to our algorithm is $\bar{E(P,Q)}=0.285$. For GDMDBClustering [25], we get $\bar{E(P,Q)}=0.285$ when the learning rate η=0.0005 or η=0.001, and the error increases to $\bar{E(P,Q)}=0.29$ when η=0.002. The average running times and clustering errors of our algorithm, GDMDBClustering [25] and BestDatabaseClustering [22] are summarized in Table A4.

Our algorithm and GDMDBClustering [25] terminate once we reach the global minimum of the convergence test function L(θ). Consequently, the running times of our algorithm and GDMDBClustering [25] are most of the time shorter than that of BestDatabaseClustering [22]. Overall, the running time of GDMDBClustering [25] stays relatively steady with respect to *n*. However, GDMDBClustering depends strongly on the learning step size η and its decay rate. On the other hand, our algorithm is learning-rate-free and needs at most (in the worst case) $(n^{2}-n)/2$ iterations to converge. Consequently, our proposed algorithm is faster than both BestDatabaseClustering [22] and GDMDBClustering [25].

To illustrate the statistically significant superiority of the proposed clustering model in terms of running time and clustering accuracy, we have applied the Friedman test [52] (under a significance level α=0.05) on the measurements (execution times and clustering errors depicted in Figure A8, Figure A9 and Figure A10) obtained by our algorithm, BestDatabaseClustering [22] and GDMDBClustering [25] (with three different values for the learning rate η) considering all the random samples in Table 7.

After conducting the Friedman test [52], we obtained the results shown in Table A5, Table A6 and Table A7, namely the average running time, the average clustering error $\bar{E(P,Q)}$ (22), the standard deviation (SD), the variance (Var), the critical value (stat) and its *p*-value for all the tested clustering algorithms, considering all 91 random samples generated via scikit-learn [50].

We notice that all the obtained results in Table A5, Table A6 and Table A7 show *p*-values that are below the significance level α=0.05. Consequently, the test suggests a rejection of the null hypothesis, stating that the compared clustering models have a similar performance. In fact, the proposed clustering algorithm significantly outperforms the other compared models, as it has the shortest average running time (6.367 milliseconds) and the lowest average clustering error ($\bar{E(P,Q)}=0.285$) among all the compared models.

### 4.5. Clustering Comparison and Assessment

In the third part of our experiments, we are interested in using some information retrieval measures to compare the clusterings produced by our algorithm and some other clustering algorithms with the ground-truth data.

Let $D=\{D_{1},D_{2},\ldots ,D_{n}\}$ be *n* transactional databases. Let $P=\{P_{1},P_{2},\ldots ,P_{k}\}$ be a *k*-class clustering of D produced by any given clustering algorithm, and let $Q=\{Q_{1},Q_{2},\ldots ,Q_{l}\}$ be the ground-truth clustering of the databases in D, such that $\cup_{i=1}^{k}\left\{P_{i}\right\}=\cup_{i=1}^{l}\left\{Q_{i}\right\}=D$ and $\cap_{i=1}^{k}\left\{P_{i}\right\}=\cap_{i=1}^{l}\left\{Q_{i}\right\}=\emptyset$. Let us define $Pairs_{P}$ and $Pairs_{Q}$ as the set of database pairs obtained from each cluster of the same clustering. That is, $Pairs_{P}=\cup_{P_{t}\in P}\cup_{D_{r},D_{s}\in P_{t};r<s}\left\{\left(D_{r},D_{s}\right)\right\}$ and $Pairs_{Q}=\cup_{Q_{t}\in Q}\cup_{D_{r},D_{s}\in Q_{t};r<s}\left\{\left(D_{r},D_{s}\right)\right\}$. To compare the two clusterings P with Q, few methods method [53,54,55] could be used. In this paper, we use a pair counting [56,57,58,59] to calculate some information retrieval measures [60,61], including precision, recall, F-measure (i.e., harmonic mean of recall and precision), Rand index [62] and Jaccard index [63] over pairs of databases being clustered together in P and/or Q. This will allow us to assess whether the predicted database pairs from P cluster together in Q, i.e., are the discovered database pairs in $Pairs_{P}$ correct with respect to the underlying true pairs in $Pairs_{Q}$ from the ground-truth clustering Q.

In Table A9, we show the categories of database pairs which represent the working set of all pair counting measures cited in Table A10. Precisely, **a:** represents the number of pairs that exist in both clusterings Q and P, **d:** represents the number of pairs that do not exist in either clustering, **b:** is the number of pairs present only in clustering Q, and **c:** is the number of pairs present only in clustering P. By counting the pairs in each category, we get an indicator for agreement and disagreement of the two clusterings being compared. The following example illustrates how to compute the measures defined in Table A10 for two given clusterings $P=\{\left\{D_{1},D_{2},D_{3}\right\},\left\{D_{4},D_{5},D_{6},D_{7}\right\}\}$ and the ground-truth partitioning $Q=\{\left\{D_{1},D_{2},D_{3}\right\},\left\{D_{4}\right\},\left\{D_{5},D_{6},D_{7}\right\}\}$ of seven transaction databases $D=\cup_{i=1}^{7}\left\{D_{i}\right\}$. First, let us calculate the following pairing categories:

$$\begin{array}{cc} Pairs_{D}= & \cup_{r=1}^{6}\cup_{s=r+1}^{7}\left\{\left(D_{r},D_{s}\right)\right\},\\ Pairs_{Q}= & \{\left(D_{6},D_{7}\right),\left(D_{5},D_{7}\right),\left(D_{5},D_{6}\right),\left(D_{2},D_{3}\right),\left(D_{1},D_{3}\right),\left(D_{1},D_{2}\right)\},\\ Pairs_{P}= & \{\left(D_{6},D_{7}\right),\left(D_{5},D_{7}\right),\left(D_{5},D_{6}\right),\left(D_{4},D_{7}\right),\left(D_{4},D_{6}\right),\left(D_{4},D_{5}\right),\left(D_{2},D_{3}\right),\left(D_{1},D_{3}\right),\\ & (D_{1},D_{2})\}. \end{array}$$

Then, $a=|Pairs_{Q}\cap Pairs_{P}|=6$, $b=|Pairs_{Q}\backslash Pairs_{P}|=0$, $c=|Pairs_{P}\backslash Pairs_{Q}|=3$, $d=|Pairs_{D}\backslash \left(Pairs_{Q}\cup Pairs_{P}\right)|=12$. Therefore, we get the following measures: F-measure=0.8, precision=0.66, recall=1.0, Rand=0.857, Jaccard=0.66. We note that the higher the values of the evaluation measures given in Table A10, the better the matching of the clustering P to its corresponding ground-truth clustering Q.

In Table A8 and Table A11, we report the F-measure [60,61], precision [60,61], recall [60,61], Rand [62] and Jaccard [63] reached by the clustering algorithms in [21,22,23], and our proposed algorithm on the datasets shown in Figure A1, Figure A2, Figure A3, Figure A4, Figure A5, Figure A6, and Figure A7. From Table A8 and Table A11, we notice that our algorithm achieves the best scores against the compared clustering algorithms, considering all the experiments in Figure A1, Figure A2, Figure A3, Figure A4, Figure A5, Figure A6, and Figure A7.

## 5. Conclusions

An improved similarity-based clustering algorithm for multi-database mining was proposed in this paper. Unlike the previous works, our algorithm requires fewer upper-bounded iterations to minimize a convex clustering quality measure. In addition, we have proposed a preprocessing layer prior to clustering where the pairwise similarities between multiple databases are first adjusted to reduce their fuzziness. This will help the clustering process to be more precise and less confused in discriminating between the different database clusters. To assess the performance of our algorithm, we conducted several experiments on real and synthetic datasets. Compared to the existing clustering algorithms for multi-database mining, our algorithm achieved the best performance in terms of accuracy and running time. In this paper, we have used the most frequent itemsets mined from each transaction database as feature sets to compute the pairwise similarities between the multiple databases. However, when the sizes of these input vectors become large, building the similarity matrix will increase the CPU overhead drastically. Moreover, the existence of some noisy frequent itemsets (FIs) may largely influence how databases are clustered together. In future work, we will investigate the impact of compressing the size of the FIs into a latent variable represented in a lower dimensional space with discriminative features. Practically, reconstituting the input vectors from the embedding space using deep auto-encoders and non-linear dimensionality reduction techniques, such as T-SNE (t-distributed stochastic neighbor embedding) and UMAP (uniform manifold approximation and projection), will force the removal of the noisy features present in the input data while keeping only the meaningful discriminative ones. Consequently, this may help improve the accuracy and running time of the clustering algorithm. Additionally, we are interested in exploring new ways to reduce the computational time used to calculate the similarity matrix via locality sensitive hashing (LSH) techniques, such as BagMinHash for weighted sets. These methods aim to encode the feature-set vectors into hash-code signatures in order to efficiently estimate the Jaccard similarity between the local transactional databases. Last but not least, in order to design a parallel version of the proposed algorithm, we will study and explore some high-performance computing tools, such as MapReduce and Spark, as an attempt to improve the clustering performance for multi-database mining.

## Figures and Tables

**Table 1:** Six transactional databases $D_{p}$, for p=1,…,6.

Transactional Database $\left(D_{p}\right)$	Transactions/Rows
$D_{1}$	(A,C),(A,B,C),(B,C),(A,B,C,D)
$D_{2}$	(A,B,C),(B,C),(A,B),(A,C),(A,B,D)
$D_{3}$	(B,C),(A,D),(B,C,D),(A,B,C)
$D_{4}$	(E,F,H),(F,H),(F,G,H,I,J)
$D_{5}$	(E,J),(F,H,J),(E,F,H,J),(F,H)
$D_{6}$	(E,I),(E,F,H),(F,H,I,J),(E,H,J)

**Table 2:** The frequent itemsets (FIs) discovered from each transactional database in Table 1 under a threshold α=0.5.

Transactional Database $\left(D_{p}\right)$	Frequent Itemsets $\mathrm{FIS}(D_{p},\alpha )$
$D_{1}$	〈AC,0.75〉,〈AB,0.5〉,〈ABC,0.5〉,〈BC,0.75〉,〈C,1.0〉,〈B,0.75〉,〈A,0.75〉
$D_{2}$	〈AB,0.6〉,〈C,0.6〉,〈B,0.8〉,〈A,0.8〉
$D_{3}$	〈BC,0.75〉,〈D,0.5〉,〈C,0.75〉,〈B,0.75〉,〈A,0.5〉
$D_{4}$	〈H,1.0〉,〈F,1.0〉,〈FH,1.0〉
$D_{5}$	〈E,0.5〉,〈EJ,0.5〉,〈J,0.75〉,〈HJ,0.5〉,〈FHJ,0.5〉,〈FJ,0.5〉,〈H,0.75〉,〈FH,0.75〉,〈F,0.75〉
$D_{6}$	〈I,0.5〉,〈J,0.5〉,〈HJ,0.5〉,〈F,0.5〉,〈FH,0.5〉,〈E,0.75〉,〈EH,0.5〉,〈H,0.75〉

**Table 3:** A summary of the clustering quality measures mentioned in this paper.

Clustering Quality [Reference]	Function (Equation)	Optimal Value
[20]	goodness(D)=B(D)+W(D)−f(D) $\left\{\begin{array}{c} B\left(D\right)=\sum_{C_{t},C_{v}\in C;t<v}\sum_{D_{p}\in C_{t},D_{q}\in C_{v};p<q}\left(1-sim\left(D_{p},D_{q}\right)\right)\\ W\left(D\right)=\sum_{C_{t}\in C}\sum_{D_{p},D_{q}\in C_{t};p<q}sim\left(D_{p},D_{q}\right)\times 𝟙\left\{\left(D_{p},D_{q}\right)\in E\right\}\\ f(D):\mathrm{number}\;\mathrm{of}\;\mathrm{clusters}. \end{array}\right.$	$$\begin{array}{c} \max\;goodness(D) \end{array}$$
[23]	${\mathrm{goodness}}^{2}\left(D\right)=\frac{\mathrm{sum}-\mathrm{dist}(D)}{(n^{2}-n)/2}+\frac{\mathrm{coupling}(D)}{(n^{2}-n)/2}+\frac{f(D)-1}{n-1}$ $\left\{\begin{array}{c} sum-dist\left(D\right)=\sum_{C_{t}\in C}\sum_{D_{p},D_{q}\in C_{t};p<q}\left(1-sim\left(D_{p},D_{q}\right)\right)\times 𝟙\left\{\left(D_{p},D_{q}\right)\in E\right\}\\ coupling\left(D\right)=\sum_{C_{t},C_{v}\in C;t<v}\sum_{D_{p}\in C_{t},D_{q}\in C_{v};p<q}sim\left(D_{p},D_{q}\right) \end{array}\right.$	$$\begin{array}{c} \min\;goodness^{2}\left(D\right) \end{array}$$
[21]	${\mathrm{goodness}}^{3}\left(D\right)=\frac{\mathrm{intra}-\mathrm{sim}(D)+\mathrm{inter}-\mathrm{dist}(D)}{f(D)}$ $\left\{\begin{array}{c} intra-sim\left(D\right)=\frac{1}{f(D)}\sum_{C_{t}\in C}\left\{\begin{array}{c} 1,\;|C_{t}|=1\\ \frac{\sum_{D_{p},D_{q}\in C_{t}}sim\left(D_{p},D_{q}\right)\times 𝟙\left\{\left(D_{p},D_{q}\right)\in E\right\}}{(|C_{t}{|}^{2}-|C_{t}|)/2},\left|C_{t}\right|>1 \end{array}\right.\\ inter-dist\left(D\right)=\left\{\begin{array}{c} 0,\;f(D)=1\\ \sum_{C_{t},C_{v}\in C}\frac{2\times \sum_{D_{p}\in C_{t},D_{q}\in C_{v};p<q}\left(1-sim\left(D_{p},D_{q}\right)\right)}{|C_{t}\left|\times \right|C_{v}|\times \left(f{\left(D\right)}^{2}-f\left(D\right)\right)},f\left(D\right)>1 \end{array}\right. \end{array}\right.$	$$\begin{array}{c} \max\;goodness^{3}\left(D\right) \end{array}$$
[43,44]	$\mathrm{SC}\left(D\right)=\frac{1}{n}\sum_{p=0}^{n-1}s\left(D_{p}\right)$ $\left\{\begin{array}{c} s\left(D_{p}\right)=\left\{\begin{array}{c} \frac{b\left(D_{p}\right)-a\left(D_{p}\right)}{\max\{a\left(D_{p}\right),b\left(D_{p}\right)\}}\;,\;\left|C_{p}\right|>1;\\ 0,\;if\;|C_{p}|=1 \end{array}\right.\\ \left\{\begin{array}{c} a\left(D_{p}\right)=\frac{\sum_{D_{p},D_{q}\in C_{p},p<q}\left(1-sim\left(D_{p},D_{q}\right)\right)\times 𝟙\left\{\left(D_{p},D_{q}\right)\in E\right\}}{|C_{p}-1|}\\ b\left(D_{p}\right)=\min_{D_{p}\notin C_{q}}\frac{1}{|C_{q}|}\sum_{D_{q}\in C_{q}}\left(1-sim\left(D_{p},D_{q}\right)\right) \end{array}\right. \end{array}\right.$	$$\begin{array}{c} \max\;SC(D) \end{array}$$

**Table 4:** Clustering the three databases $D_{1}$, $D_{2}$ and $D_{3}$ under the similarity measure simi [20] against our proposed measure sim (3).

Output	Clustering 1 under *simi* [20]	Clustering 2 under *sim* (3)
*clusters*	$\left\{D_{1}\right\},\left\{D_{2},D_{3}\right\}$	$\left\{D_{1},D_{2}\right\},\left\{D_{3}\right\}$
Similarity *intra-cluster*	0.6	0.75
Distance *inter-cluster*	1.6	1.75
Measure *goodness* [20]	0.2	0.5

**Table 5:** Itemsets synthesized from $C_{2,3}=\left\{D_{2},D_{3}\right\}$ discovered under simi [20] against the itemsets synthesized from $C_{1,2}=\left\{D_{1},D_{2}\right\}$ discovered under sim (3).

Synthesized Itemsets $I_{k}$	$\mathrm{supp}\;(I_{k},C_{2,3})$ under *simi* [20]	$\mathrm{supp}\;(I_{k},C_{1,2})$ under *sim* (3)
*A*	$0.12<\alpha_{2,3}=0.19$	$0.2>\alpha_{1,2}=0.17$
*B*	$0.12<\alpha_{2,3}=0.19$	$0.2>\alpha_{1,2}=0.17$
*C*	$0.12<\alpha_{2,3}=0.19$	$0.2>\alpha_{1,2}=0.17$
*E*	$0.9>\alpha_{2,3}=0.19$	$0.54>\alpha_{1,2}=0.17$

**Table 6:** A summary of the results obtained in Figure 5, Figure 6, Figure 7 and Figure 8. We note that $\delta_{\mathrm{opt}}$ is the optimal similarity level at which goodness(D) [20] attains its maximum value, and $\theta^{T}$ is the optimal weight vector learned after a number of epochs.

Similarity Matrix	Fuzziness Index (9)	$\theta^{T},\mathrm{epochs},\eta$	max goodness(D) [20]	$\delta_{\mathrm{opt}}$	SC(D) [43,44] at $\delta_{\mathrm{opt}}$	Optimal Clustering at $\delta_{\mathrm{opt}}$
Figure 5	0.97	$\theta^{T}=\left[1,1,\ldots ,1,1\right]$ (Without fuzziness reduction)	4.19	0.46	−1	$\left\{D_{1},D_{2},D_{3},D_{4},D_{5}\right\}$
Figure 5	0.95	$\theta^{T}=\left[1,1,\ldots ,1,1\right]$ (Without fuzziness reduction)	1.29	0.313	−1	$\left\{D_{1},D_{2},D_{3},D_{4}\right\}$
Figure 6	0.74	$\theta^{T}$ = [1.30,0.52,0.71,0.71,0.52, 0.71,0.71,0.52,0.52,1.44], epochs=300, η=0.1	4.54	0.95	0.73	$\left\{D_{1},D_{2}\right\},\left\{D_{3}\right\},\left\{D_{4},D_{5}\right\}$
Figure 6	0.81	$\theta^{T}=\left[0.63,0.638,0.591,0.712,0.712,0.77\right]$, epochs=100, η=0.1	1.27	0.292	0.08	$\left\{D_{4},D_{3},D_{2}\right\},\left\{D_{1}\right\}$

**Table 7:** A brief summary of the random blobs generated via scikit-learn [50].

Number of Random Blobs (n)	Number of Centers $\left\lfloor \frac{n}{2}\right\rfloor$	Number of Attributes (m)
30	15	random.randint(2, 10)
⋮	⋮	⋮
60	30	random.randint(2, 10)
⋮	⋮	⋮
120	60	random.randint(2, 10)

## Data Availability

All datasets are available at http://fimi.ua.ac.be/data/ (accessed on 25 April 2021) and https://archive.ics.uci.edu/ml/datasets (accessed on 25 April 2021).

## Abbreviations

The following abbreviations are used in this manuscript:

FIs	Frequent Itemsets
FIM	Frequent Itemset Mining
MDB	Multiple Databases
MDM	Multi-database Mining
CD	Coordinate Descent
CL	Competitive Learning
BMU	Best Matching Unit
T-SNE	t-Distributed Stochastic Neighbor Embedding
UMAP	Uniform Manifold Approximation and Projection
LSH	Locality Sensitive Hashing

## Appendix A

**Table A1:** Description of the datasets used in our experiments with their database partitions and the number frequent itemsets (FIs) mined from each database partition $D_{i}$ and the number of FIs mined from each cluster $C_{j}$ under a threshold α.

Dataset Name/Ref	Number of Rows	Number of Rows in Partition $D_{i}$	Number of $\mathrm{FIS}(D_{i},\alpha )$ from Partition $D_{i}$	Number of FIS(D,α) from Dataset D	Ground Truth Clustering	Number of $\mathrm{FIS}(C_{j},\alpha )$ from Cluster $C_{j}$
Mushroom [48] (2 classes)	8124	$|D_{1}|=3916$ ($C_{1}$) $|D_{2}|=1402$ ($C_{2}$) $|D_{3}|=1402$ ($C_{2}$) $|D_{4}|=1404$ ($C_{2}$)	$|FIS(D_{1},0.5)|=375$ $|FIS(D_{2},0.5)|=2063$ $|FIS(D_{3},0.5)|=32,911$ $|FIS(D_{4},0.5)|=807$	|FIS(D,0.5)|=151	$C_{1}=\left\{D_{1}\right\},$ $C_{2}=\left\{D_{4},D_{3},D_{2}\right\}$	$|FIS(C_{1},0.5)|=375$ $|FIS(C_{2},0.5)|=1441$
Zoo [48] (7 classes)	101	$|D_{1}|=20$ ($C_{1}$) $|D_{2}|=21$ ($C_{1}$) $|D_{3}|=10$ ($C_{2}$) $|D_{4}|=10$ ($C_{2}$) $|D_{5}|=5$ ($C_{3}$) $|D_{6}|=6$ ($C_{4}$) $|D_{7}|=7$ ($C_{4}$) $|D_{8}|=2$ ($C_{5}$) $|D_{9}|=2$ ($C_{5}$) $|D_{10}|=4$ ($C_{6}$) $|D_{11}|=4$ ($C_{6}$) $|D_{12}|=10$ ($C_{7}$)	$|FIS(D_{1},0.5)|=24,383$ $|FIS(D_{2},0.5|=30,975$ $|FIS(D_{3},0.5)|=30,719$ $|FIS(D_{4},0.5)|=32,767$ $|FIS(D_{5},0.5)|=20,479$ $|FIS(D_{6},0.5|=65,535$ $|FIS(D_{7},0.5)|=65,535$ $|FIS(D_{8},0.5)|=114,687$ $|FIS(D_{9},0.5)|=98,303$ $|FIS(D_{10},0.5)|=53,247$ $|FIS(D_{11},0.5)|=57,343$ $|FIS(D_{12},0.5)|=28,671$	|FIS(D,0.5)|=0	$C_{1}=\left\{D_{2},D_{1}\right\},$ $C_{2}=\left\{D_{4},D_{3}\right\},$ $C_{3}=\left\{D_{5}\right\},$ $C_{4}=\left\{D_{7},D_{6}\right\},$ $C_{5}=\left\{D_{9},D_{8}\right\},$ $C_{6}=\left\{D_{11},D_{10}\right\},$ $C_{7}=\left\{D_{12}\right\},$	$|FIS(C_{1},0.5)|=25,087$ $|FIS(C_{2},0.5)|=28,671$ $|FIS(C_{3},0.5)|=2479$ $|FIS(C_{4},0.5)|=49,151$ $|FIS(C_{5},0.5)|=57,343$ $|FIS(C_{6},0.5)|=45,055$ $|FIS(C_{7},0.5)|=28,671$
Iris [48] (3 classes)	150	$|D_{1}|=25$ ($C_{1}$) $|D_{2}|=25$ ($C_{1}$) $|D_{3}|=25$ ($C_{2}$) $|D_{4}|=25$ ($C_{2}$) $|D_{5}|=25$ ($C_{3}$) $|D_{6}|=25$ ($C_{3}$)	$|FIS(D_{1},0.2)|=5$ $|FIS(D_{2},0.2)|=6$ $|FIS(D_{3},0.2)|=2$ $|FIS(D_{4},0.2)|=2$ $|FIS(D_{5},0.2)|=2$ $|FIS(D_{6},0.2)|=5$	|FIS(D,0.2)|=0	$C_{1}=\left\{D_{2},D_{1}\right\},$ $C_{2}=\left\{D_{4},D_{3}\right\},$ $C_{3}=\left\{D_{6},D_{5}\right\}$	$|FIS(C_{1},0.2)|=3$ $|FIS(C_{2},0.2)|=1$ $|FIS(C_{3},0.2)|=2$
T10I4D100K [49] (unknown classes)	100,000	$|D_{i}|=10,000$ rows, i=1…10	$|FIS(D_{1},0.03)|=58$ $|FIS(D_{2},0.03)|=58$ $|FIS(D_{3},0.03)|=62$ $|FIS(D_{4},0.03)|=57$ $|FIS(D_{5},0.03)|=62$ $|FIS(D_{6},0.03)|=63$ $|FIS(D_{7},0.03)|=63$ $|FIS(D_{8},0.03)|=59$ $|FIS(D_{9},0.03)|=61$ $|FIS(D_{10},0.03)|=62$	|FIS(D,0.03)|=50	Seven clusters found via the silhouette coefficient [43] $C_{1}=\left\{D_{1}\right\},$ $C_{2}=\left\{D_{2}\right\},$ $C_{3}=\left\{D_{3}\right\},$ $C_{4}=\left\{D_{5},D_{4}\right\},$ $C_{5}=\left\{D_{6}\right\},$ $C_{6}=\left\{D_{7}\right\},$ $C_{7}=\left\{D_{10},D_{9},D_{8}\right\}$	$|FIS(C_{1},0.03)|=58$ $|FIS(C_{2},0.03)|=58$ $|FIS(C_{3},0.03)|=62$ $|FIS(C_{4},0.03)|=59$ $|FIS(C_{5},0.03)|=63$ $|FIS(C_{6},0.03)|=59$ $|FIS(C_{7},0.03)|=61$
Figure A1 [20] (unknown classes)	24	$|D_{1}|=3$ $|D_{2}|=3$ $|D_{3}|=3$ $|D_{4}|=4$ $|D_{5}|=4$ $|D_{6}|=3$ $|D_{7}|=4$	$|FIS(D_{1},0.42)|=3$ $|FIS(D_{2},0.42)|=3$ $|FIS(D_{3},0.42)|=5$ $|FIS(D_{4},0.42)|=7$ $|FIS(D_{5},0.42)|=7$ $|FIS(D_{6},0.42)|=5$ $|FIS(D_{7},0.42)|=3$	|FIS(D,0.42)|=0	Three clusters found via the silhouette coefficient [43] $C_{1}=\left\{D_{3},D_{2},D_{1}\right\}$ $C_{2}=\left\{D_{4}\right\}$ $C_{3}=\left\{D_{7},D_{6},D_{5}\right\}$	$|FIS(C_{1},0.42)|=3$ $|FIS(C_{2},0.42)|=7$ $|FIS(C_{3},0.42)|=3$

**Table A2:** Clustering results illustrated in Figure A1, Figure A2, Figure A3, Figure A4, Figure A5, Figure A6, and Figure A7 after using the clustering quality measures ${\mathrm{goodness}}^{3}\left(D\right)$ [21], ${\mathrm{goodness}}^{2}\left(D\right)$ [23], the silhouette coefficient SC(D) [43], goodness(D) [20] and our proposed objective function L(θ), where δ is the level of similarity at which each clustering evaluation/loss function reaches its optimal value.

Dataset Name/Ref	Silhouette Coefficient	Clustering Result Using Proposed Objective		Clustering Result/ Optimal Value		Clustering Result/ Optimal Value		Clustering Result/ Optimal Value	
	max SC(D)	*Clusters*	$L(\arg\min_{\begin{array}{c} \theta \end{array}}L\left(\theta \right))$	Clusters	max goodness(D)	Clusters	min ${\mathrm{goodness}}^{2}\left(D\right)$	Clusters	max ${\mathrm{goodness}}^{3}\left(D\right)$
Figure A1 7×7 [20]	0.46 at δ=0.444	$C_{1}=\left\{D_{3},D_{2},D_{1}\right\},$ $C_{2}=\left\{D_{4}\right\},$ $C_{3}=\left\{D_{7},D_{6},D_{5}\right\}$	2.004 at δ=0.444	$C_{1}=\left\{D_{3},D_{2},D_{1}\right\},$ $C_{2}=\left\{D_{4}\right\},$ $C_{3}=\left\{D_{7},D_{6},D_{5}\right\}$	15.407 at δ=0.444	$C_{1}=\left\{D_{7},\ldots ,D_{1}\right\}$	0.259 at δ=0.065	$C_{1}=\left\{D_{3},D_{2},D_{1}\right\},$ $C_{2}=\left\{D_{7},\ldots ,D_{4}\right\}$	0.728 at δ=0.086
Figure A2 12×12 Zoo [48]	0.41 at δ=0.559	$C_{1}=\left\{D_{2},D_{1}\right\},$ $C_{2}=\left\{D_{4},D_{3}\right\},$ $C_{3}=\left\{D_{5}\right\},$ $C_{4}=\left\{D_{7},D_{6}\right\},$ $C_{5}=\left\{D_{9},D_{8}\right\},$ $C_{6}=\left\{D_{11},D_{10}\right\},$ $C_{7}=\left\{D_{12}\right\}$	7.71 at δ=0.559	$C_{1}=\left\{D_{2},D_{1}\right\},$ $C_{2}=\left\{D_{4},D_{3}\right\},$ $C_{3}=\left\{D_{5}\right\},$ $C_{4}=\left\{D_{7},D_{6}\right\},$ $C_{5}=\left\{D_{9},D_{8}\right\},$ $C_{6}=\left\{D_{11},D_{10}\right\},$ $C_{7}=\left\{D_{12}\right\}$	32.98 at δ=0.559	$C_{1}=\left\{D_{12},\ldots ,D_{1}\right\}$	0.57 at δ=0.348	$C_{1}=\left\{D_{12},\ldots ,D_{1}\right\}$	0.42 at δ=0.348
Figure A3 4×4 Mushroom [48]	0.08 at δ=0.41	$C_{1}=\left\{D_{1}\right\},$ $C_{2}=\left\{D_{4},D_{3},D_{2}\right\}$	0.43 at δ=0.41	$C_{1}=\left\{D_{4},\ldots ,D_{1}\right\}$	1.672 at δ=0.365	$C_{1}=\left\{D_{4},\ldots ,D_{1}\right\}$	0.55 at δ=0.365	$C_{1}=\left\{D_{1}\right\},$ $C_{2}=\left\{D_{4},D_{3},D_{2}\right\}$	0.68 at δ=0.41
Figure A4 6×6 Iris [48]	0.304 at δ=0.3	$C_{1}=\left\{D_{2},D_{1}\right\},$ $C_{2}=\left\{D_{4},D_{3}\right\},$ $C_{3}=\left\{D_{6},D_{5}\right\}$	1.10 at δ=0.3	$C_{1}=\left\{D_{2},D_{1}\right\},$ $C_{2}=\left\{D_{4},D_{3}\right\},$ $C_{3}=\left\{D_{6},D_{5}\right\}$	9.64 at δ=0.3	$C_{1}=\left\{D_{2},D_{1}\right\},$ $C_{2}=\left\{D_{4},D_{3}\right\},$ $C_{3}=\left\{D_{6},D_{5}\right\}$	0.55 at δ=0.3	$C_{1}=\left\{D_{2},D_{1}\right\},$ $C_{2}=\left\{D_{4},D_{3}\right\},$ $C_{3}=\left\{D_{6},D_{5}\right\}$	0.44 at δ=0.3
Figure A5 6×6 Zoo & Mushroom [48]	0.63 at δ=0.384	$C_{1}=\left\{D_{2},D_{1}\right\},$ $C_{2}=\left\{D_{6},\ldots ,D_{3}\right\}$	0.5 at δ=0.384	$C_{1}=\left\{D_{2},D_{1}\right\},$ $C_{2}=\left\{D_{6},\ldots ,D_{3}\right\}$	9.96 at δ=0.384	$C_{1}=\left\{D_{2},D_{1}\right\},$ $C_{2}=\left\{D_{6},\ldots ,D_{3}\right\}$	0.40 at δ=0.384	$C_{1}=\left\{D_{2},D_{1}\right\},$ $C_{2}=\left\{D_{6},\ldots ,D_{3}\right\}$	0.85 at δ=0.384
Figure A6 4×4 [39]	0.34 at δ=0.429	$C_{1}=\left\{D_{3},D_{2},D_{1}\right\},$ $C_{2}=\left\{D_{4}\right\}$	1.12 at δ=0.429	$C_{1}=\left\{D_{4},\ldots ,D_{1}\right\}$	2.708 at δ=0.25	$C_{1}=\left\{D_{4},\ldots ,D_{1}\right\}$	0.38 at δ=0.25	$C_{1}=\left\{D_{3},D_{2},D_{1}\right\},$ $C_{2}=\left\{D_{4}\right\}$	0.81 at δ=0.429
Figure A7 10×10 T10I4D100K [49]	0.115 at δ=0.846	$C_{1}=\left\{D_{1}\right\},$ $C_{2}=\left\{D_{2}\right\},$ $C_{3}=\left\{D_{3}\right\},$ $C_{4}=\left\{D_{5},D_{4}\right\},$ $C_{5}=\left\{D_{6}\right\},$ $C_{6}=\left\{D_{7}\right\},$ $C_{7}=\left\{D_{10},D_{9},D_{8}\right\}$	0.71 at δ=0.846	$C_{1}=\left\{D_{10},\ldots ,D_{1}\right\}$	35.275 at δ=0.737	$C_{1}=\left\{D_{10},\ldots ,D_{1}\right\}$	0.193 at δ=0.737	$C_{1}=\left\{D_{10},\ldots ,D_{1}\right\}$	0.806 at δ=0.737

**Table A3:** The similarity levels $\delta_{opt}$ at which the clustering evaluation measures ${\mathrm{goodness}}^{3}\left(D\right)$ [21], ${\mathrm{goodness}}^{2}\left(D\right)$ [23], the silhouette coefficient SC(D) [43], goodness(D) [20] and our proposed objective function L(θ) attain their optimal values in Figure A1, Figure A2, Figure A3, Figure A4, Figure A5, Figure A6, and Figure A7. The fraction $\frac{\left|\{\delta_{1},\ldots ,\delta_{\mathrm{stop}}\}\right|}{\left|\{\delta_{1},\ldots ,\delta_{m}\}\right|}$ represents the number of similarity levels required to test the convergence and terminate divided by the number of total similarity levels *m*.

Dataset D	Silhouette Coefficient $\max_{\begin{array}{c} \theta \end{array}}SC\left(D\right)$		Proposed Loss Function $L(\arg\min_{\begin{array}{c} \theta \end{array}}L\left(\theta \right))$		Goodness Measure $\max_{\theta }\mathrm{goodness}\left(D\right)$		Goodness Measure $\min_{\theta }{\mathrm{goodness}}^{2}\left(D\right)$		Goodness Measure $\max_{\theta }{\mathrm{goodness}}^{3}\left(D\right)$	
	$\delta_{\mathrm{opt}}$	$\frac{\left|\{\delta_{1},\ldots ,\delta_{\mathrm{stop}}\}\right|}{\left|\{\delta_{1},\ldots ,\delta_{m}\}\right|}$	$\delta_{\mathrm{opt}}$	$\frac{\left|\{\delta_{1},\ldots ,\delta_{\mathrm{stop}}\}\right|}{\left|\{\delta_{1},\ldots ,\delta_{m}\}\right|}$	$\delta_{\mathrm{opt}}$	$\frac{\left|\{\delta_{1},\ldots ,\delta_{\mathrm{stop}}\}\right|}{\left|\{\delta_{1},\ldots ,\delta_{m}\}\right|}$	$\delta_{\mathrm{opt}}$	$\frac{\left|\{\delta_{1},\ldots ,\delta_{\mathrm{stop}}\}\right|}{\left|\{\delta_{1},\ldots ,\delta_{m}\}\right|}$	$\delta_{\mathrm{opt}}$	$\frac{\left|\{\delta_{1},\ldots ,\delta_{\mathrm{stop}}\}\right|}{\left|\{\delta_{1},\ldots ,\delta_{m}\}\right|}$
Figure A1 7×7 [20]	0.444	$\frac{10}{10}$	0.444	$\frac{5}{10}$	0.444	$\frac{10}{10}$	0.065	$\frac{10}{10}$	0.086	$\frac{10}{10}$
Figure A2 12×12 Zoo [48]	0.559	$\frac{48}{48}$	0.559	$\frac{5}{48}$	0.559	$\frac{48}{48}$	0.348	$\frac{48}{48}$	0.348	$\frac{48}{48}$
Figure A3 4×4 Mushroom [48]	0.41	$\frac{4}{4}$	0.41	$\frac{2}{4}$	0.365	$\frac{4}{4}$	0.365	$\frac{4}{4}$	0.41	$\frac{4}{4}$
Figure A4 6×6 Iris [48]	0.3	$\frac{6}{6}$	0.3	$\frac{3}{6}$	0.3	$\frac{6}{6}$	0.3	$\frac{6}{6}$	0.3	$\frac{6}{6}$
Figure A5 6×6 Zoo & Mushroom [48]	0.384	$\frac{8}{8}$	0.384	$\frac{7}{8}$	0.384	$\frac{8}{8}$	0.384	$\frac{8}{8}$	0.384	$\frac{8}{8}$
Figure A6 4×4 [39]	0.429	$\frac{6}{6}$	0.429	$\frac{3}{6}$	0.25	$\frac{6}{6}$	0.25	$\frac{6}{6}$	0.429	$\frac{6}{6}$
Figure A7 10×10 T10I4D100K [49]	0.846	$\frac{31}{31}$	0.846	$\frac{4}{31}$	0.737	$\frac{31}{31}$	0.737	$\frac{31}{31}$	0.737	$\frac{31}{31}$

**Table A4:** Summary of the average running times and the average clustering errors $\bar{E(P,Q)}$ (22) for the proposed algorithm, BestDatabaseClustering [22] and GDMDBClustering [25] (with three different values for the learning rate η) on the random samples described in Table 7.

Experiment (Figure)	Proposed Algo		BestDatabaseClustering [22]		GDMDBClustering [25]	
	Average Running Time	Average Clustering Error	Average Running Time	Average Clustering Error	Average Running Time	Average Clustering Error
Figure A8 η=0.001	6.367	0.285	47.208	0.936	14.825	0.285
Figure A9 η=0.002	6.367	0.285	47.208	0.936	7.305	0.290
Figure A10 η=0.0005	6.367	0.285	47.208	0.936	28.479	0.285

**Table A5:** Statistical Friedman test results [52] for the measurements obtained by all the compared clustering algorithms in Figure A8.

Algorithm	Measurements									
	**Running Time**					**Clustering Error**				
	**Average**	**SD**	**Var**	**Stat**	***p*** **-Value**	**Average**	**SD**	**Var**	**Stat**	***p*** **-Value**
**Proposed Algo**	6.367	3.018	9.107			0.285	0.080	0.006		
**BestDatabaseClustering [22]**	47.208	27.537	758.313	135.707	3.40e–30	0.936	0.066	0.004	150	2.67e–33
**GDMDBClustering [25] (η=0.001)**	14.825	1.743	3.037			0.285	0.080	0.006		

**Table A6:** Statistical Friedman test results [52] for the measurements obtained by all the compared clustering algorithms in Figure A9.

Algorithm	Measurements									
	**Running Time**					**Clustering Error**				
	**Average**	**SD**	**Var**	**Stat**	***p*** **-Value**	**Average**	**SD**	**Var**	**Stat**	***p*** **-Value**
**Proposed Algo**	6.367	3.018	9.107			0.285	0.080	0.006		
**BestDatabaseClustering [22]**	47.208	27.537	758.313	121.62	3.88e–27	0.936	0.066	0.004	131	3.56e–29
**GDMDBClustering [25] (η=0.002)**	7.305	1.766	3.118			0.290	0.086	0.007		

**Table A7:** Statistical Friedman test results [52] for the measurements obtained by all the compared clustering algorithms in Figure A10.

Algorithm	Measurements									
	**Running Time**					**Clustering Error**				
	**Average**	**SD**	**Var**	**Stat**	***p*** **-Value**	**Average**	**SD**	**Var**	**Stat**	***p*** **-Value**
**Proposed Algo**	6.367	3.018	9.107			0.285	0.080	0.006		
**BestDatabaseClustering [22]**	47.208	27.537	758.313	118.90	1.51e–26	0.936	0.066	0.004	150	2.67e–33
**GDMDBClustering [25] (η=0.0005)**	28.479	4.655	21.669			0.285	0.080	0.006		

**Table A8:** F-measure [60,61], precision [60,61] and recall [60,61] reached by the compared clustering algorithms in [21,22,23], and our proposed algorithm on the datasets shown in Figure A1, Figure A2, Figure A3, Figure A4, Figure A5, Figure A6, and Figure A7. Notice that our algorithm gets the best scores for all the datasets.

Dataset	F-Measure [60,61]				Precision [60,61]				Recall [60,61]			
	Proposed Algo	Algo [22]	Algo [23]	Algo [21]	Proposed Algo	Algo [22]	Algo [23]	Algo [21]	Proposed Algo	Algo [22]	Algo [23]	Algo [21]
Figure A1 7 × 7 [20]	1	1	0.44	0.8	1	1	0.28	0.66	1	1	1	1
Figure A2 12 × 12 Zoo [48]	1	1	0.14	0.14	1	1	0.075	0.075	1	1	1	1
Figure A3 4 × 4 Mushroom [48]	1	0.66	0.66	1	1	0.5	0.5	1	1	1	1	1
Figure A4 6 × 6 Iris [48]	1	1	1	1	1	1	1	1	1	1	1	1
Figure A5 6 × 6 Zoo & Mushroom [48]	1	1	1	1	1	1	1	1	1	1	1	1
Figure A6 4 × 4 [39]	1	0.66	0.66	1	1	0.5	0.5	1	1	1	1	1
Figure A7 10 × 10 T10I4D100K [49]	1	0.16	0.16	0.16	1	0.088	0.088	0.088	1	1	1	1

**Table A9:** Contingency matrix showing the categories in pairing clustered databases.

Clustering	Predicted Clusters	
	Pairs in P	Pairs *Not* in P
**Actual clusters**		
**Pairs in** Q	$a:=|Pairs_{Q}\cap Pairs_{P}|$ (True Positive)	$b:=|Pairs_{Q}\backslash Pairs_{P}|$ (False Negative)
**Pairs not in** Q	$c:=|Pairs_{P}\backslash Pairs_{Q}|$ (False Positive)	d:= Pairs *in none* (True Negative)

**Table A10:** Pair counting measures used for clustering assessment and comparison.

Precision [60,61]	Recall [60,61]	F-Measure [60,61]	Rand [62]	Jaccard [63]
$\frac{\left(a\right)}{\left(a)+(c\right)}$	$\frac{\left(a\right)}{\left(a)+(b\right)}$	$\frac{2(a)}{2(a)+(b)+(c)}$	$\frac{\left(a)+(d\right)}{\left(a)+(b)+(c)+(d\right)}$	$\frac{\left(a\right)}{\left(a)+(b)+(c\right)}$

**Table A11:** Rand index [62] and Jaccard index [63] reached by the clustering algorithms in [21,22,23] and our proposed algorithm on the datasets shown in Figure A1, Figure A2, Figure A3, Figure A4, Figure A5, Figure A6, and Figure A7. Notice that our algorithm gets the best scores for all the datasets.

Dataset	Rand [62]				Jaccard [63]			
	Proposed Algo	Algo [22]	Algo [23]	Algo [21]	Proposed Algo	Algo [22]	Algo [23]	Algo [21]
Figure A1 7×7 [20]	1	1	0.28	0.85	1	1	0.28	0.66
Figure A2 12×12 Zoo [48]	1	1	0.075	0.075	1	1	0.075	0.075
Figure A3 4×4 Mushroom [48]	1	0.5	0.5	1	1	0.5	0.5	1
Figure A4 6×6 Iris [48]	1	1	1	1	1	1	1	1
Figure A5 6×6 Zoo & Mushroom [48]	1	1	1	1	1	1	1	1
Figure A6 4×4 [39]	1	0.5	0.5	1	1	0.5	0.5	1
Figure A7 10×10 T10I4D100K [49]	1	0.088	0.088	0.088	1	0.088	0.088	0.088

## References

[1] Han J., Pei J., Yin Y., Mao R. (2004). Mining frequent patterns without candidate generation: A frequent-pattern tree approach. Data Min. Knowl. Discov..

[2] Ng A.Y., Jordan M.I., Weiss Y. (2001). On spectral clustering: Analysis and an algorithm. Adv. Neural Inf. Process. Syst..

[3] Johnson S.C. (1967). Hierarchical clustering schemes. Psychometrika.

[4] MacQueen J. Some methods for classification and analysis of multivariate observations. Proceedings of the Fifth Berkeley Symposium on Mathematical Statistics and Probability.

[5] Zhang Y.J., Liu Z.Q. (2002). Self-splitting competitive learning: A new on-line clustering paradigm. IEEE Trans. Neural Netw..

[6] Yair E., Zeger K., Gersho A. (1992). Competitive learning and soft competition for vector quantizer design. IEEE Trans. Signal Process..

[7] Hofmann T., Buhmann J.M. (1998). Competitive learning algorithms for robust vector quantization. IEEE Trans. Signal Process..

[8] Kohonen T. (2012). Self-Organizing Maps.

[9] Pal N.R., Bezdek J.C., Tsao E.K. (1993). Generalized clustering networks and Kohonen’s self-organizing scheme. IEEE Trans. Neural Netw..

[10] Mao J., Jain A.K. (1996). A self-organizing network for hyperellipsoidal clustering (HEC). Trans. Neural Netw..

[11] Anderberg M.R. (2014). Cluster Analysis for Applications: Probability and Mathematical Statistics: A Series of Monographs and Textbooks.

[12] Aggarwal C.C., Reddy C.K. (2014). Data clustering. Algorithms and Application.

[13] Wang C.D., Lai J.H., Philip S.Y. (2013). NEIWalk: Community discovery in dynamic content-based networks. IEEE Trans. Knowl. Data Eng..

[14] Wang Z., Zhang D., Zhou X., Yang D., Yu Z., Yu Z. (2013). Discovering and profiling overlapping communities in location-based social networks. IEEE Trans. Syst. Man Cybern. Syst..

[15] Huang D., Lai J.H., Wang C.D., Yuen P.C. (2016). Ensembling over-segmentations: From weak evidence to strong segmentation. Neurocomputing.

[16] Shi J., Malik J. (2000). Normalized cuts and image segmentation. IEEE Trans. Pattern Anal. Mach. Intell..

[17] Zhao Q., Wang C., Wang P., Zhou M., Jiang C. (2016). A novel method on information recommendation via hybrid similarity. IEEE Trans. Syst. Man Cybern. Syst..

[18] Symeonidis P. (2015). ClustHOSVD: Item recommendation by combining semantically enhanced tag clustering with tensor HOSVD. IEEE Trans. Syst. Man Cybern. Syst..

[19] Rafailidis D., Daras P. (2012). The TFC model: Tensor factorization and tag clustering for item recommendation in social tagging systems. IEEE Trans. Syst. Man Cybern. Syst..

[20] Adhikari A., Adhikari J. (2015). Clustering Multiple Databases Induced by Local Patterns. Advances in Knowledge Discovery in Batabases.

[21] Liu Y., Yuan D., Cuan Y. (2013). Completely Clustering for Multi-databases Mining. J. Comput. Inf. Syst..

[22] Miloudi S., Hebri S.A.R., Khiat S. (2018). Contribution to Improve Database Classification Algorithms for Multi-Database Mining. J. Inf. Proces. Syst..

[23] Tang H., Mei Z. A Simple Methodology for Database Clustering. Proceedings of the 5th International Conference on Computer Engineering and Networks.

[24] Wang R., Ji W., Liu M., Wang X., Weng J., Deng S., Gao S., Yuan C.A. (2018). Review on mining data from multiple data sources. Pattern Recognit. Lett..

[25] Miloudi S., Wang Y., Ding W. (2021). A Gradient-Based Clustering for Multi-Database Mining. IEEE Access.

[26] Miloudi S., Wang Y., Ding W. An Optimized Graph-based Clustering for Multi-database Mining. Proceedings of the 2020 IEEE 32nd International Conference on Tools with Artificial Intelligence (ICTAI).

[27] Zhang S., Zaki M.J. (2006). Mining Multiple Data Sources: Local Pattern Analysis. Data Min. Knowl. Discov..

[28] Adhikari A., Rao P.R. (2008). Synthesizing heavy association rules from different real data sources. Pattern Recognit. Lett..

[29] Adhikari A., Adhikari J. (2015). Advances in Knowledge Discovery in Databases.

[30] Adhikari A., Jain L.C., Prasad B. (2017). A State-of-the-Art Review of Knowledge Discovery in Multiple Databases. J. Intell. Syst..

[31] Zhang S., Zhang C., Wu X. (2004). Identifying Exceptional Patterns. Knowl. Discov. Multiple Datab..

[32] Zhang S., Zhang C., Wu X. (2004). Identifying High-vote Patterns. Knowl. Discov. Multiple Datab..

[33] Ramkumar T., Srinivasan R. (2008). Modified algorithms for synthesizing high-frequency rules from different data sources. Knowl. Inf. Syst..

[34] Djenouri Y., Lin J.C.W., Nørvåg K., Ramampiaro H. Highly efficient pattern mining based on transaction decomposition. Proceedings of the 2019 IEEE 35th International Conference on Data Engineering (ICDE).

[35] Savasere A., Omiecinski E.R., Navathe S.B. (1995). An Efficient Algorithm for Mining Association Rules in Large Databases.

[36] Zhang S., Wu X. (2001). Large scale data mining based on data partitioning. Appl. Artif. Intel..

[37] Zhang C., Liu M., Nie W., Zhang S. (2004). Identifying Global Exceptional Patterns in Multi-database Mining. IEEE Intell. Inform. Bull..

[38] Zhang S., Zhang C., Yu J.X. (2004). An efficient strategy for mining exceptions in multi-databases. Inf. Sci..

[39] Wu X., Zhang C., Zhang S. (2005). Database classification for multi-database mining. Inf. Syst..

[40] Li H., Hu X., Zhang Y. (2009). An Improved Database Classification Algorithm for Multi-database Mining. Frontiers in Algorithmics.

[41] Na S., Xumin L., Yong G. Research on k-means clustering algorithm: An improved k-means clustering algorithm. Proceedings of the 2010 Third International Symposium on Intelligent Information Technology and Security Informatics.

[42] Selim S.Z., Ismail M.A. (1984). K-means-type algorithms: A generalized convergence theorem and characterization of local optimality. IEEE Trans. Pattern Anal. Mach. Intel..

[43] Rousseeuw P.J. (1987). Silhouettes: A graphical aid to the interpretation and validation of cluster analysis. J. Comput. Appl. Math..

[44] Kaufman L., Rousseeuw P.J. (2009). Finding Groups in Data: An Introduction to Cluster Analysis.

[45] De Luca A., Termini S., Dubois D., Prade H., Yager R.R. (1993). A Definition of a Nonprobabilistic Entropy in the Setting of Fuzzy Sets Theory. Readings in Fuzzy Sets for Intelligent Systems.

[46] Boyd S., Vandenberghe L. (2004). Convex Optimization.

[47] Cormen T.H., Leiserson C.E., Rivest R.L., Stein C. (2009). Data structures for disjoint sets. Introduction to Algorithms.

[48] Center for Machine Learning and Intelligent Systems UCI Machine Learning Repository. https://archive.ics.uci.edu/ml/datasets/.

[49] IBM Almaden Quest Research Group Frequent Itemset Mining Dataset Repository. http://fimi.ua.ac.be/data/..

[50] Thirion G., Varoquaux A., Gramfort V., Michel O., Grisel G., Louppe J. Nothman. Scikit-learn: Sklearn.datasets.makeblobs. https://scikit-learn.org/stable/modules/generated/sklearn.datasets.make_blobs.html.

[51] Gramfort A., Blondel M., Grisel O., Mueller A., Martin E., Patrini G., Chang E. Scikit-Learn: Sklearn.preprocessing.MinMaxScaler. https://scikit-learn.org/stable/modules/generated/sklearn.preprocessing.MinMaxScaler.html.

[52] Friedman M. (1940). A comparison of alternative tests of significance for the problem of m rankings. Ann. Math. Stat..

[53] Meilǎ M. Comparing clusterings: An axiomatic view. Proceedings of the 22nd International Conference on Machine Learning.

[54] Vinh N.X., Epps J., Bailey J. (2010). Information theoretic measures for clusterings comparison: Variants, properties, normalization and correction for chance. J. Mach. Learn. Res..

[55] Günnemann S., Färber I., Müller E., Assent I., Seidl T. External evaluation measures for subspace clustering. Proceedings of the 20th ACM International Conference on Information and Knowledge Management.

[56] Banerjee A., Krumpelman C., Ghosh J., Basu S., Mooney R.J. Model-based overlapping clustering. Proceedings of the Eleventh ACM SIGKDD International Conference on Knowledge Discovery in Data Mining.

[57] Pfitzner D., Leibbrandt R., Powers D. (2009). Characterization and evaluation of similarity measures for pairs of clusterings. Knowl. Inf. Syst..

[58] Achtert E., Goldhofer S., Kriegel H.P., Schubert E., Zimek A. Evaluation of clusterings–metrics and visual support. Proceedings of the 2012 IEEE 28th International Conference on Data Engineering.

[59] Shafiei M., Milios E. Model-based overlapping co-clustering. Proceedings of the SIAM Conference on Data Mining.

[60] Chinchor N. MUC-4 evaluation metrics. Proceedings of the of the Fourth Message Understanding Conference.

[61] Mei Q., Radev D. (1979). Information retrieval. The Oxford Handbook of Computational Linguistics.

[62] Rand W.M. (1971). Objective criteria for the evaluation of clustering methods. J. Am. Stat. Assoc..

[63] Jaccard P. (1901). Distribution de la flore alpine dans le bassin des Dranses et dans quelques régions voisines. Bull. Soc. Vaudoise Sci. Nat..

